# UBTOR/KIAA1024 regulates neurite outgrowth and neoplasia through mTOR signaling

**DOI:** 10.1371/journal.pgen.1007583

**Published:** 2018-08-06

**Authors:** Hefei Zhang, Quan Zhang, Ge Gao, Xinjian Wang, Tiantian Wang, Zhitao Kong, Guoxiang Wang, Cuizhen Zhang, Yun Wang, Gang Peng

**Affiliations:** Institute of Brain Science, State Key Laboratory of Medical Neurobiology and Collaborative Innovation Center for Brain Science, Fudan University, Shanghai, China; Brigham and Women's Hospital, UNITED STATES

## Abstract

The mTOR signaling pathways regulate cell growth and are involved in multiple human diseases. Here, we identify *UBTOR*, a previously unannotated gene as a functional player in regulating cell growth and mTOR signaling. Reduction of UBTOR function in cultured hippocampal neurons and PC12 cells promotes neurite outgrowth. UBTOR depletion activates mTOR signaling and promotes cell growth, whilst UBTOR overexpression suppresses colony formation in cancer cell lines. Studies in cultured cells and zebrafish model show that UBTOR inhibits mTOR signaling by stabilizing the mTOR complex component DEPTOR, and *ubtor* gene disruption result in higher mTOR activity and aggravate HRAS(G12V) induced neoplasia in the zebrafish. Lastly, UBTOR depletion promotes tumor growth and mTOR signaling in a xenograft mouse model. Together, our results demonstrate how UBTOR regulates cell growth and neoplasia via mTOR signaling.

## Author summary

Cell growth is a fundamental aspect of cell behavior in all organisms. The mTOR signaling pathways are essential for cell growth and clinically mis-regulation of the mTOR pathways are implicated in human diseases including tumor formation, obesity, epilepsy, autism and neurodegeneration. Here, we identify a novel gene, *Ubtor* as a functional player in regulating cell growth and mTOR signaling. Inhibiting *Ubtor* function promotes cell growth in neurons and cancer cells. Increasing *Ubtor* function reduces cancer cell growth. Functional analyses in human cells and the zebrafish model indicate *Ubtor* inhibits mTOR signaling by stabilizing the mTOR complex component DEPTOR, and *ubtor* gene disruption resulted in higher mTOR activity and aggravated cancer formation in the zebrafish. UBTOR depletion promotes tumor growth and mTOR signaling in xenograft-bearing mice. Thus our study provide evidence that *Ubtor* constitutes a novel negative feedback mechanism to control mTOR signaling and cell growth, and manipulations of Ubtor function may potentially be utilized to optimize mTOR signaling activities for treatments of cancers and other diseases.

## Introduction

Mechanistic target of rapamycin (mTOR) signaling is a central pathway that controls cell metabolism, growth, proliferation and survival. Clinically the mTOR pathway is implicated in human diseases including tumor formation, obesity, epilepsy, autism and neurodegeneration [1–3].

mTOR signaling activity is regulated by various factors at multiple cellular organelles [4]. The central catalytic protein mTOR forms two complexes mTORC1 and mTORC2 through interactions with a number of proteins. These mTOR interacting proteins exert important regulatory roles on mTOR activity. A protein of interest is DEPTOR, a component of both mTOR complexes and an inhibitor of mTOR signaling found only in the vertebrate species [5]. DEPTOR is phosphorylated and ubiquitylated when mTORC1 signaling pathway is activated by serum stimulation, which subsequently results in its degradation [6–8].

Here, we identify *Ubtor* as a functional player in regulating cell growth and mTOR signaling. *Ubtor* is a vertebrate-specific, previously unannotated gene except being listed as a downregulated or mutated gene in tumor tissues [9–11]. *Ubtor* encodes a protein without any known functional domains. We first show reductions of Ubtor expression levels in cultured hippocampal neurons and PC12 cells promote neurite outgrowth. In addition, UBTOR depletion promotes cell growth in HEK293T and U87MG cells, whilst UBTOR overexpression suppresses colony formation in HEK293T and T24 cells. We next show reduction of Ubtor promotes mTOR signaling. Mechanistic studies in cultured cells show UBTOR interacts with DEPTOR and mTOR complexes. Further studies show UBTOR stabilizes DEPTOR and regulates DEPTOR’s ubiquitination. Lastly, *ubtor* gene disruption in zebrafish increases mTOR activity and aggravates HRAS(G12V) induced neoplasia in the intact animals. In parallel, Ubtor depletion promotes tumor growth and mTOR signaling in xenograft-bearing mice. Together, our results demonstrate how Ubtor regulates cell growth and neoplasia via mTOR signaling.

## Results

### Ubtor depletion promotes neurite outgrowth in hippocampal neurons and PC12 cells

*kiaa1024/ubtor* was identified in a zebrafish enhancer-trap screen aimed to isolate genes with expression in the central nervous system (see Materials and Methods). It was an unannotated gene and present in the vertebrate species only. In situ hybridization results showed *ubtor* was expressed in the brain and spinal cord in the zebrafish (S1 Fig). RT-PCR results showed *ubtor* was additionally expressed in other tissues including internal organs in the zebrafish. Based on expression data from the Allen Mouse Brain Atlas 12] and the GENSAT [13], *Ubtor* gene expression patterns in the nervous system appeared conserved between the zebrafish and the mouse.

**Fig 1:**
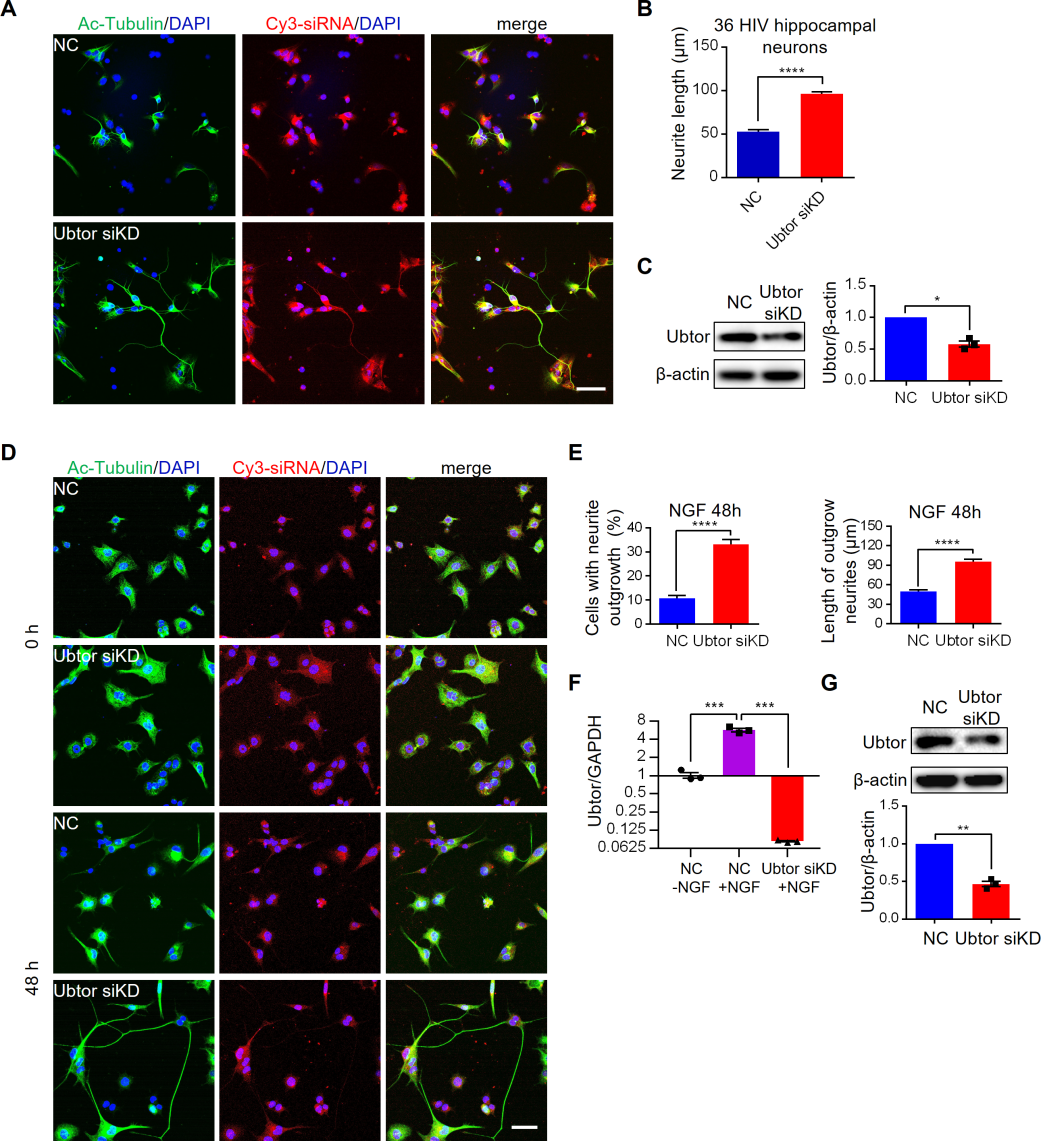
Ubtor depletion promotes neurite outgrowth in hippocampal neurons and ld-PC12 cells. (**A**) Neurite outgrowth in primary culture of rat hippocampal neurons. Dissociated hippocampal neurons were transfected with either Cy3-labeled negative control siRNA (NC) or *Ubtor* siRNA (Ubtor siKD) and then cultured in vitro for 36 hrs (HIV). Neurites were stained by the acetylated tubulin antibody. Transfected cells were indicated by the Cy3 fluorescence signals from the Cy3-labeled siRNAs. Scale bar, 50 μm. (**B**) Quantitative analysis of neurite outgrowth at 36 HIV. Neurite lengths were measured from 15 images for the NC, and 16 images for the Ubtor siKD groups, taken from 3 independent experiments. n = 241 and 288 for the NC and the Ubtor siKD group, respectively. *t* = 12.71, *df* = 527, *P* < 0.0001. (**C**) Immunoblot analysis of siRNA mediated knock down in hippocampal neurons. *t* = 8.427, *df* = 2, *P* = 0.0138. (**D**) NGF-induced neurite outgrowths in ld-PC12 cells transfected with either Cy3-labeled negative control siRNA (NC) or *Ubtor* siRNA (Ubtor siKD). Transfected cells were serum-starved overnight and treated with 50 ng/ml of NGF for 0 and 48 hours. Scale bar, 50 μm. (**E**) Quantitative analysis of neurite outgrowth at 48 hours post NGF treatment. Neurite outgrowth rates were calculated from 38 images for the NC, and 36 images for the Ubtor siKD groups, taken from four independent experiments. *t* = 9.827, *df* = 72, *P* < 0.0001. Neurite lengths of differentiated cells were measured in these images. *n* = 88 and 243 for the NC and the *Ubtor* siKD group, respectively. *t* = 7.721, *df* = 329, *P* < 0.0001. (**F**) qRT-PCR analysis of Ubtor expression levels. Expression levels relative to GAPDH levels are normalized to the untreated NC group. Three biological repeats, F_(2, 6)_ = 181.1, *P* < 0.0001. Multiple comparison significance values are indicated on the graph. (**G**) Immunoblot analysis of siRNA mediated knock down in ld-PC12 cells. *t* = 14.94, *df* = 2, *P* < 0.01.

To investigate the function of *Ubtor*, we examined neurite outgrowth in rat primary hippocampal neurons (Fig 1). Fluorescence dye Cy3 labeled small interference RNA (Cy3-siRNA) was transfected into the dissociated hippocampal neurons to knock down *Ubtor* expression levels (Fig 1C), and the outgrowth of the neurites were revealed by acetylated tubulin stain and measured at 36 hours post in vitro culture (HIV) and 56 HIV (Fig 1A and S2A Fig). The results showed the neurite outgrowth length was almost twice long in *Ubtor* knock-down hippocampal neurons compared with neurons transfected with control siRNA at 36 HIV (Fig 1B). Longer neurite outgrowth length was also observed in *Ubtor* knock-down hippocampal neurons at 56 HIV (S2A Fig).

We next examined the nerve growth factor (NGF) induced neurite outgrowth in PC12 cells [14]. We tested both PC12 cells and a PC12 subline (ld-PC12), in which the expression level of *Ubtor* was higher than that in the PC12 cell line (S2D Fig). Interestingly, NGF treatment increased *Ubtor* mRNA levels by about six-fold in the ld-PC12 cells (Fig 1F). Small interference RNA (siRNA) was transfected into the ld-PC12 cells to knock down *Ubtor* expression levels (Fig 1G). After the transfected cells were treated with NGF for 48 hours, the neurite outgrowth length doubled in *Ubtor* knock-down cells compared with cells transfected with control siRNA (Fig 1D and 1E). Enhanced neurite outgrowth was also observed when *Ubtor* function was inhibited in the PC12 cells (S2B Fig).

### UBTOR regulates cell growth

**Fig 2:**
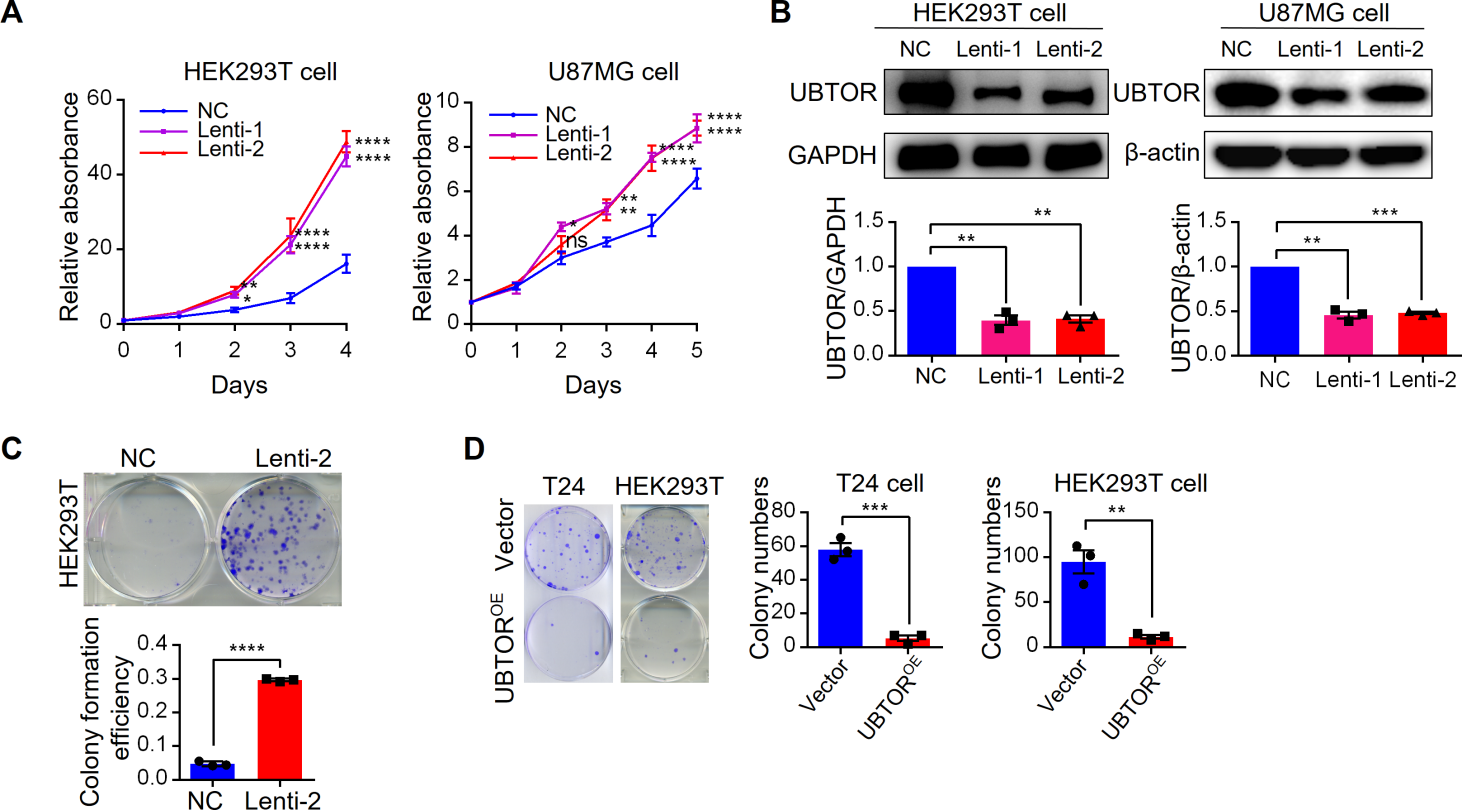
Depletion of UBTOR promotes cell growth. (**A**) Cell proliferation analyses of HEK293T and glioma U87MG cells transfected with negative control (NC) or UBTOR Lentiviral shRNAs (Lenti-1 or Lenti-2). Three biological repeats for each cell line. For the Lentiviral shRNA treatment factor, HEK293T: F_(2, 6)_ = 116.1, *P* < 0.0001, and U87MG: F_(2, 6)_ = 11.10, *P* < 0.0096. Multiple comparison significance values are indicated on the graph. (**B**) Immunoblot analysis of shRNA mediated knock down in HEK293T and U87MG cells. Three biological repeats for each cell line. For the Lentiviral shRNA treatment factor, HEK293T: F_(2, 6)_ = 81.41, *P* < 0.0001, and U87MG: F_(2, 6)_ = 180.4, *P* < 0.0001. Multiple comparison significance values are indicated on the graph. (**C**) Colony formation analysis of HEK293T cells with reduced UBTOR expression (Lenti-2) and control cells (NC). Transfected cells were plated at 500 cells/well in 6-well plate and grew for 7 days. Three biological repeats, *t* = 49.98, *df* = 4, *P* < 0.0001. (**D**) Colony formation analysis of T24 and HEK293T cells transfected with vector control (Vector) or UBTOR over-expressing construct UBTOR-FLAG-HA (UBTOR^OE^). Transfected cells were plated at 5 x 10^4^ (T24) or 2 x 10^4^ (HEK293T) cells/plate in 6 cm plates and grew for 2 weeks. Three biological repeats, *t* = 12.73, *df* = 4, *P* < 0.001 for the T24 cells and *t* = 6.383, *df* = 4, *P* < 0.01 for the HEK293T cells.

*UBTOR* was listed as a downregulated or mutated gene in tumor tissues in previous studies [9–11]. Expression analyses in human cancer samples by the Xena Browser (http://xena.ucsc.edu) and the FireBrowse (http://firebrowse.org/) tools showed *UBTOR* was markedly downregulated in adrenocortical cancer, pheochromocytoma and paraganglioma, and glioma (S3 Fig). Thus we examined effects of *UBTOR* reduction in cultured human cells by lentivirus mediated shRNA knockdowns (Fig 2B). The results showed *UBTOR* knock-down promoted proliferation in human HEK293T cells and human glioblastoma U87MG cells (Fig 2A). In addition, *UBTOR* knock-down also promoted colony formation in HEK293T cells (Fig 2C). In contrast, overexpression of *UBTOR* decreased colony formation in both the carcinoma T24 cells and the HEK293T cells (Fig 2D).

### Ubtor depletion promotes mTOR signaling

In PC12 cells, NGF mainly acts through TrkA receptor to promote neurite outgrowth. Activated TrkA receptor functions through two major downstream signaling pathways: Ras-MAPK and PI3K pathways [15–18]. We found no difference in the phosphorylation levels of ERK1/2, a crucial kinase of the Ras-MAPK signaling pathway, between the *Ubtor* knock-down and control PC12 cells (S4B Fig).

**Fig 3:**
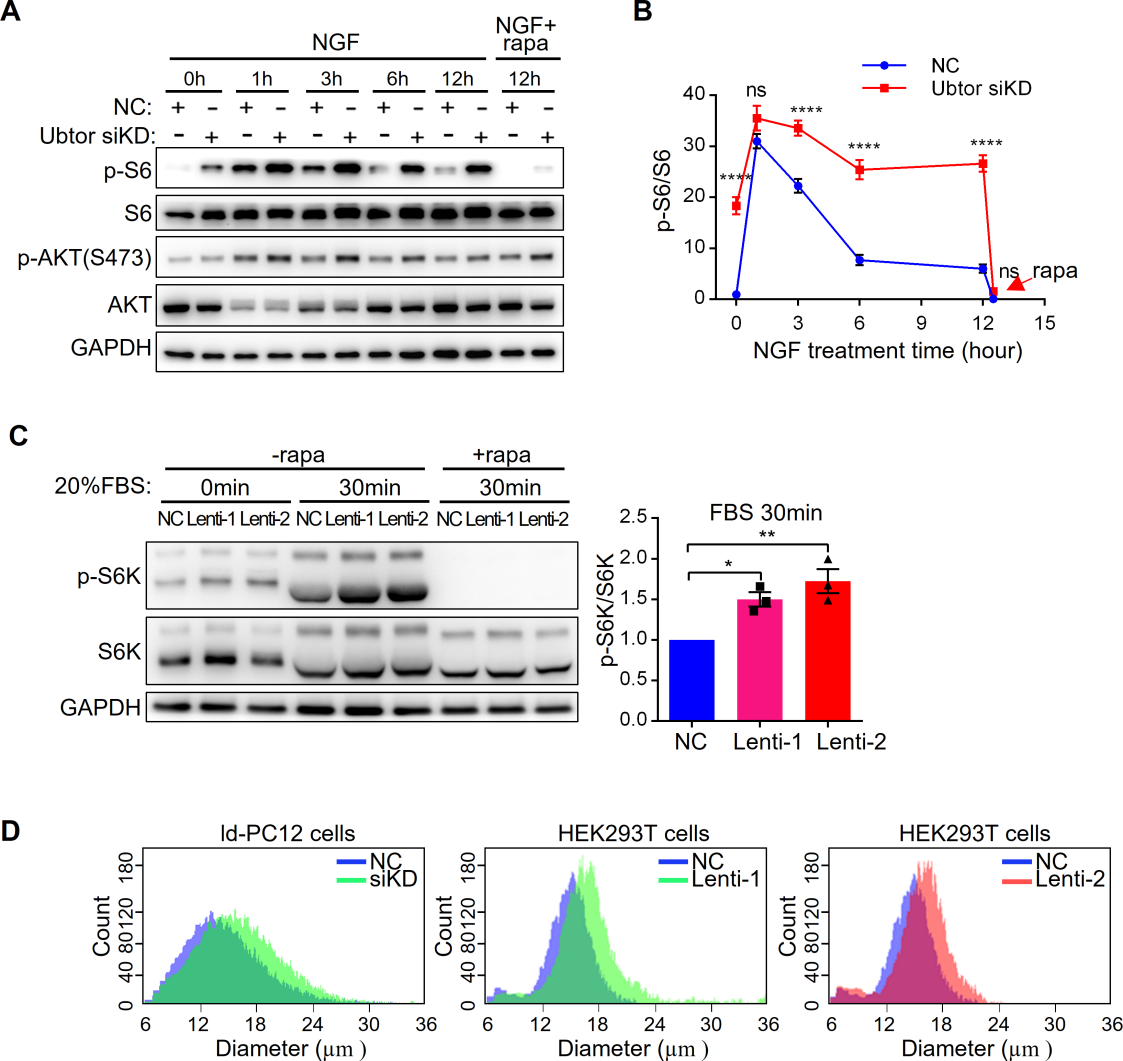
Ubtor depletion increases mTOR signaling. (**A**) Immunoblot analysis of mTOR signaling pathway in ld-PC12 cells transfected with either negative control siRNA (NC) or *Ubtor* siRNA (Ubtor siKD). Transfected cells were serum starved overnight and treated with 50 ng/ml of NGF for 0 to 12 hours. In addition, cells were treated with 100 nM of rapamycin (rapa) or vehicle (DMSO) for 30 min after 12 hours of NGF treatment. GAPDH was used as a loading control. Representative results from four biological repeats. (**B**) Quantitative analysis of p-S6 levels. The p-S6/S6 values were normalized to that of the NC group. Four biological repeats. For the siRNA treatment factor, F_(1, 6)_ = 88.21, *P* < 0.0001. Multiple comparison significance values across different time points are indicated on the graph. (**C**) Immunoblot analysis of mTOR signaling pathway in HEK293T cells transfected with Lentiviral shRNAs to reduce UBTOR expression. Transfected cells were serum starved overnight and then treated with 20% serum (FBS) with or without 100 nM of rapamycin for 30 min. The p-S6K/S6K values were normalized to that of the NC group. Three biological repeats. For the Lentiviral shRNA treatment factor, F_(2, 6)_ = 13.66, *P* < 0.01. (**D**) ld-PC12 or HEK293T cells with reduced UBTOR expression were larger than control cells. Cell diameter distributions were measured in ld-PC12 or HEK293T cells transfected with negative control (NC) or siRNA (siKD) or shRNA (Lenti-1 or Lenti-2). Representative results from three biological repeats.

One important downstream target of PI3K is the mTOR complex. Phosphorylation level of RPS6 (p-S6), a read-out of mTORC1 activity, was marked higher in the *Ubtor* knock-down ld-PC12 cells before NGF treatment (Fig 3A). One or 3 hours after NGF was added, the p-S6 levels were increased in both the *Ubtor* knock-down and control cells, with higher levels in the *Ubtor* knock-down cells. With longer NGF treatment at 6 hours and 12 hours, the p-S6 level decreased in control cells, while in *Ubtor* knock-down cells the high p-S6 level was maintained (Fig 3A and 3B). NGF-induced p-S6 up-regulation was abolished by co-treatment of mTORC1 specific inhibitor rapamycin (Fig 3A and 3B). Consistent with mTORC1 activation, the cell diameters of the ld-PC12 cells with reduced *Ubtor* expression were larger than those of the control cells (Fig 3D). In contrast to p-S6, the phosphorylation level of AKT(S473), a read-out of mTORC2 activity, was slightly higher in the *Ubtor* knock-down cells (Fig 3A). Similar increases of p-S6 levels were observed in the PC12 cells when *Ubtor* was knocked-down; and rapamycin treatment blocked S6 phosphorylation (S4A Fig).

Similar to results in the PC12 cells, the phosphorylation levels of ERK1/2 were not changed in HEK293T cells with reduced UBTOR expression (S4C Fig). Phosphorylation levels of p70 S6K (p-S6K), another read-out of mTORC1 activity, were significantly higher in *UBTOR* knock-down cells upon serum stimulation and rapamycin treatment blocked S6K phosphorylation in both the control and the *UBTOR* knock-down cells (Fig 3C). HEK293T cells with reduced UBTOR expression were also larger than control cells (Fig 3D).

### UBTOR interacts with DEPTOR

**Fig 4:**
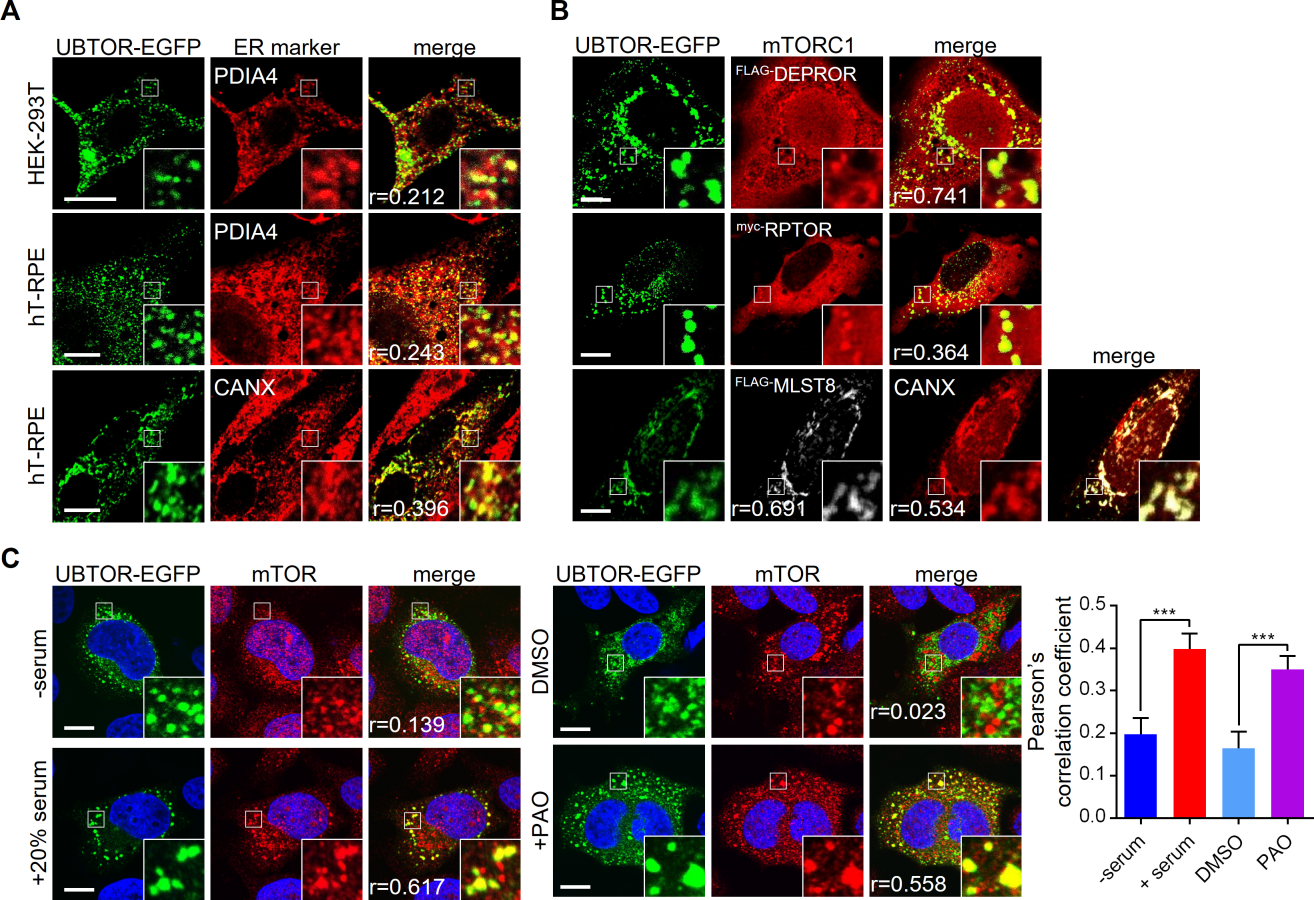
Subcellular localization of UBTOR and mTOR complexes. (**A**) Subcellular localization of UBTOR in HEK293T and hT-RPE cells, co-stained with endoplasmic reticulum (ER) marker PDIA4 or CANX. Figure inserts show enlarged views of the boxed area. Pearson correlation coefficient or Pearson’s *r* values are indicated on bottom left. Representative results from three biological repeats. Scale bar, 10 μm. (**B**) Subcellular localization of UBTOR and mTORC1 complex components in HeLa cells. Plasmid constructs encoding FLAG-tagged DEPTOR, myc-tagged RAPTOR, or FLAG-tagged MLST8 were co-transfected with EGFP-tagged UBTOR and immunostained with FLAG or myc antibodies respectively. Pearson’s *r* values are indicated on bottom left. Representative results from three biological repeats. Scale bar, 10 μm. (**C**) Serum or PAO treatment increases co-localization of UBTOR and endogenous mTOR protein. For serum treatment, transfected HeLa cells were first serum starved overnight and then treated with 20% FBS for 1 hour. For PAO treatment, transfected cells were treated with vehicle (DMSO) or 10 μM of PAO for 15 min. Quantitative analysis of co-localization is shown on the right. Representative results from 4 biological repeats. For the serum test, *t* = 3.776, *df* = 44, *P* = 0.005. For the PAO test, *t* = 3.742, *df* = 59, *P* = 0.0004. Scale bar, 10 μm.

Protein sequence analysis tools indicated UBTOR was a transmembrane protein with most amino acid residues located in the cytoplasm. Subcellular localization results confirmed UBTOR was localized to the endoplasmic reticulum and the plasma membrane, with the bulk of protein on the cytoplasm side (Fig 4A and S5 Fig).

We investigated whether UBTOR interacted with the mTOR complex. We first co-transfected HeLa cells with tagged UBTOR and mTOR components and examined their subcellular co-localization. Under basal growth condition, UBTOR co-localized with exogenously expressed DEPTOR, RPTOR, and MLST8 proteins (Fig 4B). We next validated an antibody that recognized endogenous mTOR protein (S6 Fig). We found upon serum stimulation or treatment of phenylarsine oxide (PAO), a chemical activator of mTORC1 signaling pathway [19, 20], co-localization between UBTOR and endogenous mTOR was observed (Fig 4C).

**Fig 5:**
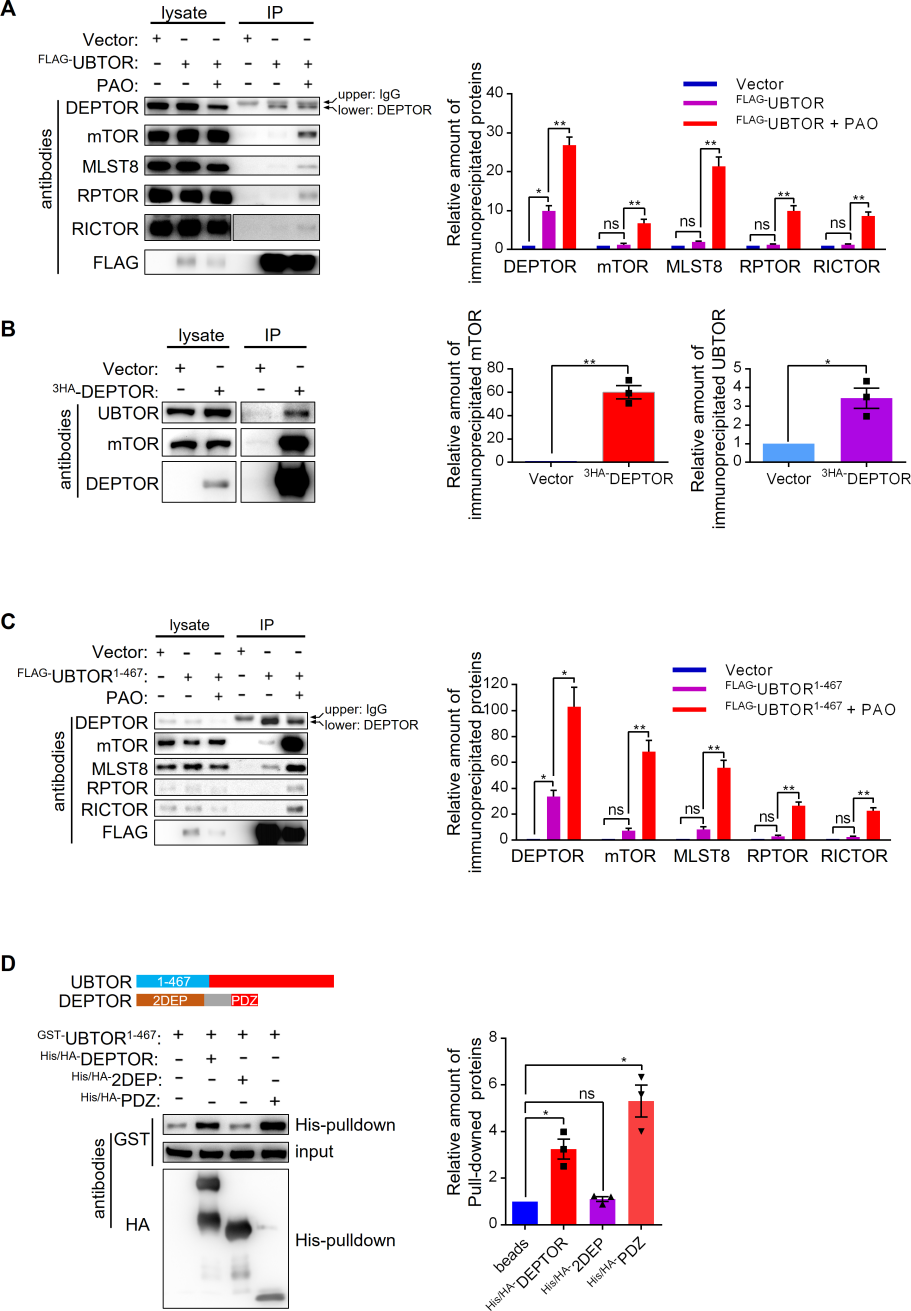
UBTOR interacts with mTOR complexes through DEPTOR. (**A**) PAO treatment promotes interactions between UBTOR and endogenous mTOR complexes. HEK293T cells transfected with FLAG-tagged UBTOR were treated with 5 μM of PAO or vehicle (DMSO) for 15 min. Cell lysates were immunoprecipitated with anti-FLAG-M2 beads, and then probed with antibodies indicated on the figure panel. UBTOR interacted with DEPTOR with or without PAO treatment. Representative results from three biological repeats. Quantitative analysis of the immunoblots is shown on the right. Statistics significance values were indicated on the graph. (**B**) HA-tagged DEPTOR immunoprecipitates endogenous UBTOR. Quantitative analysis of the immunoblots is shown on the right. For DEPTOR-mTOR interaction test, *t* = 10.28, *df* = 2, *P* = 0.0093. For DEPTOR-UBTOR interaction test, *t* = 4.473, *df* = 2, *P* = 0.0465. (**C**) The N terminal region of UBTOR (UBTOR^1-467^) is sufficient to interact with endogenous DEPTOR and endogenous mTOR complexes. Experimental procedure similar to that of **A**. Representative results from three biological repeats. Quantitative analysis of the immunoblots is shown on the right. Statistics significance values were indicated on the graph. (**D**) The N terminal region of UBTOR (UBTOR^1-467^) directly interacts with the full length DEPTOR or the PDZ domain of DEPTOR. GST-tagged UBTOR^1-467^, his/HA double tagged DEPTOR, 2DEP domain, and PDZ domain proteins were expressed and purified from bacteria. Purified proteins were mixed, pulled-down by Nickle-beads, and immunoblotted as indicated. Representative results from three biological repeats. Quantitative analysis of the immunoblots is shown on the right. Statistics significance values are indicated on the graph.

We further examined UBTOR-mTOR complex interaction by co-immunoprecipitation. Under basal growth condition, FLAG-tagged UBTOR co-precipitated with endogenously expressed DEPTOR protein, but not other components of mTOR complex (Fig 5A). Reciprocally, exogenously-expressed DEPTOR co-immunoprecipitated with endogenously expressed UBTOR (Fig 5B). Interestingly, when PAO was added to activate mTOR signaling, UBTOR co-immunopreciptations with endogenous DEPTOR, mTOR, RPTOR and MLST8 were observed (Fig 5A). Finally, we found the N terminal region of UBTOR (UBTOR^1-467^) strongly immunoprecipitated with endogenous DEPTOR under basal growth condition, and PAO treatment enhanced interactions between UBTOR^1-467^ and components of mTOR complexes (Fig 5C). Bacteria-source purified UBTOR^1-467^ directly interacted with the PDZ domain of DEPTOR (Fig 5D). These results indicated that UBTOR constitutively interacted with DEPTOR, and UBTOR may form stable interaction with the mTOR complexes under serum or PAO stimulated conditions.

### UBTOR stabilizes DEPTOR by regulating its ubiquitination level

**Fig 6:**
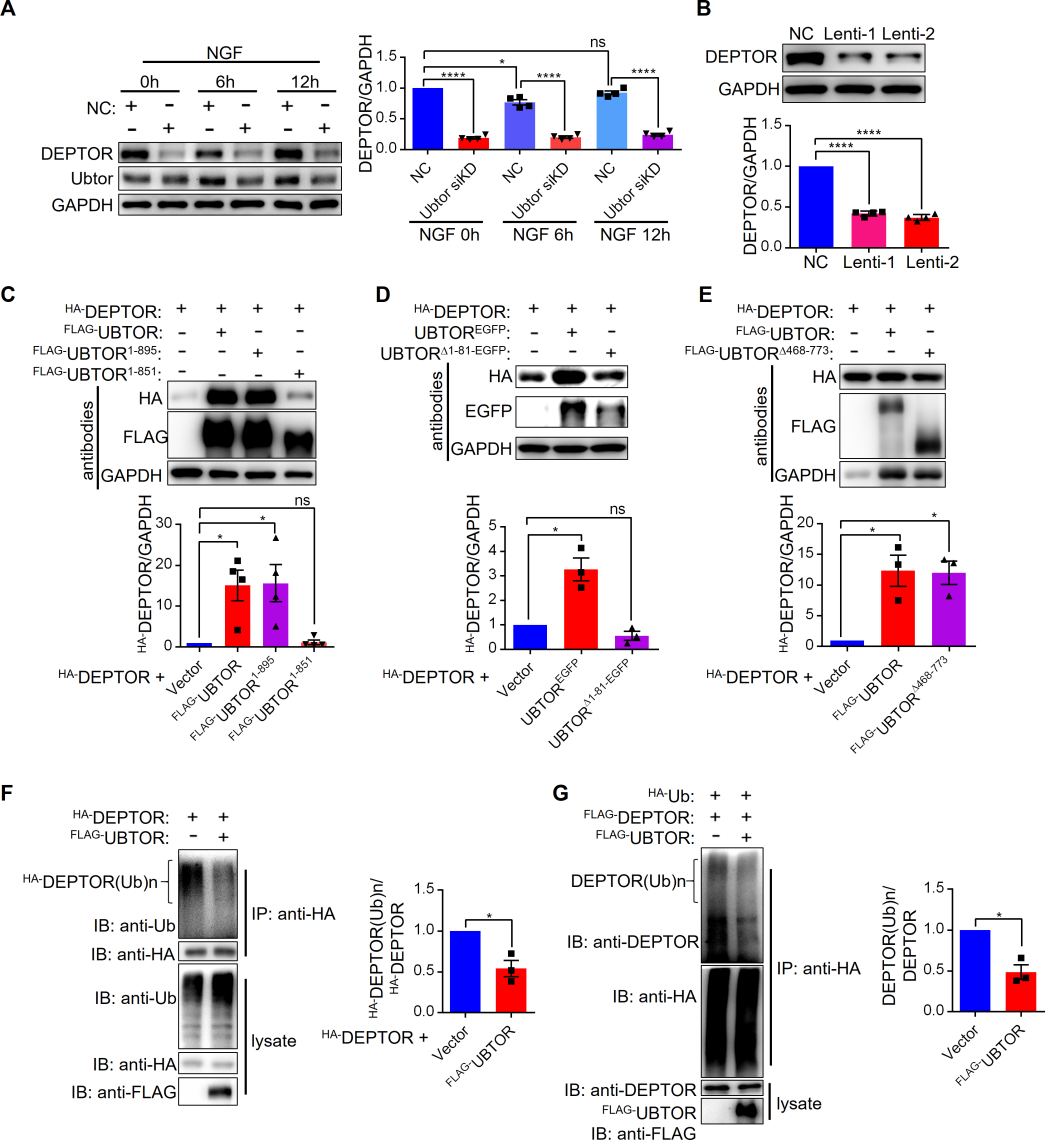
UBTOR stabilizes DEPTOR by regulating ubiquitination levels of DEPTOR. (**A**) Immunoblot analysis of DEPTOR expression levels in ld-PC12 cells transfected with negative control (NC) and Ubtor siRNA. Transfected cells were treated with 50 ng/ml of NGF for 0 to 12 hours as indicated. Representative results from four biological repeats. Quantitative analysis of DEPTOR expression levels is shown on the right. The DEPTOR/GAPDH values were normalized to that of the NC group. Four biological repeats, Statistics significance values are indicated on the graph. (**B**) Immunoblot analysis of DEPTOR expression levels in HEK293T cells transfected with negative control (NC) and Ubtor Lentiviral shRNA (Lenti-1 and Lenti-2). Transfected cells were serum-starved overnight. Quantitative analysis was same as in **A**. Four biological repeats, F_(2, 9)_ = 618.5, *P* < 0.0001. Multiple comparison significance values are indicated on the graph. (**C**) Immunoblot analysis of DEPTOR expression levels in HEK293T cells co-transfected with DEPTOR and various UBTOR constructs. UBTOR^1-895^: without transmembrane motif (Δ896–916). UBTOR^1-851^: without C terminal region (Δ852–916). Representative results from four biological repeats. Quantitative analysis of immunoblots is shown below. (**D**) The N terminal region (1–81) is required for UBTOR’s stabilization effect on DEPTOR. Representative results from three biological repeats. Quantitative analysis of immunoblots is shown below. (**E**) The middle region (468–773) of UBTOR is dispensable for UBTOR’s stabilization effect on DEPTOR. Representative results from three biological repeats. Quantitative analysis of immunoblots is shown below. (**F**) UBTOR overexpression reduces ubiquitination levels of DEPTOR in HEK293T cells. HA-tagged DEPTOR was co-transfected with either empty vector or FLAG-tagged UBTOR. Transfected cells were serum starved overnight, and then treated with 10% FBS and 20 μM MG132 for 6 hours. Cell lysates were immunoprecipitated with anti-HA beads, then probed with antibodies as indicated. Representative results from three biological repeats. Quantitative analysis of immunoblots is shown on the right. (**G**) Immunoblot analysis of DEPTOR ubiquitination levels. HEK293T cells were co-transfected as indicated. Representative results from three biological repeats. Quantitative analysis of immunoblots is shown on the right.

DEPTOR is an inhibitor of mTORC1 and mTORC2 [5, 21]. DEPTOR is phosphorylated and ubiquitylated when mTOR signaling pathway is activated by serum stimulation, which results in its degradation [6–8]. In the PC12 cells, the expression level of DEPTOR was reduced by 40% after 6 hours treatment of NGF (Fig 6A), consistent with degradation of DEPTOR caused by mTOR activation. Compare with the 40% reduction in the control cells, when UBTOR level was knocked down, the expression level of DEPTOR was reduced more than five-fold before and throughout the NGF treatment (Fig 6A). Thus, these marked reductions in UBTOR knockdown cells could not solely be due to mTOR signaling activation and subsequent DEPTOR degradation. Alternatively, UBTOR might stabilize DEPTOR by its direct interaction with DEPTOR. In agreement with UBTOR’s stabilization effect on DEPTOR, knockdown of UBTOR in HEK293T cells caused significant reduction of DEPTOR under basal growth condition (Fig 6B). To further examine this issue, we transiently expressed DEPTOR in HEK293T cells and found when UBTOR was co-expressed with DEPTOR, DEPTOR’s expression level was increased (Fig 6C). The transmembrane domain of UBTOR (Δ896–916) was dispensable for the stabilization effect on DEPTOR, whilst further deletion into the C terminal region of UBTOR protein (Δ852–916) abrogated this stabilization effect (Fig 6C). As expected, the UBTOR^1-467^ N terminal region was required for the full stabilization effects of DEPTOR (Fig 6D), because UBTOR^1-467^ interacted with DEPTOR (Fig 5). The middle region of UBTOR (468–773) was not required for the stabilization of DEPTOR (Fig 6E).

Previous studies have shown DEPTOR was degraded through ubiquitin-proteasome pathway [6–8]. Thus, UBTOR may stabilize DEPTOR by regulating ubiquitination of DEPTOR. Indeed, UBTOR reduced the ubiquitination level of DEPTOR when they were co-expressed in HEK293T cells (Fig 6F and 6G).

### *ubtor* mutation elevates mTOR signaling and aggravates neoplasia in vivo

To investigate Ubtor’s functions in intact animals, *ubtor* gene was disrupted in zebrafish by TALEN and CRISPR/Cas9 mediated mutagenesis (see Materials and Methods). The targeted gene disruption resulted in frame-shift and multiple stop codons in the Ubtor coding sequence. Thus no functional Ubtor protein could be made in the homozygous mutant. The homozygous *ubtor* mutant had no gross developmental abnormalities. They were of similar sizes as their wild type siblings and were fertile under standard raising and maintaining conditions. Results from behavior tests showed *ubtor* mutant had enhanced fear-evoked freezing and compromised C-start responses (S7 Fig), suggesting *ubtor* mutation had subtle but significant effects on neurodevelopment and animal physiology.

**Fig 7:**
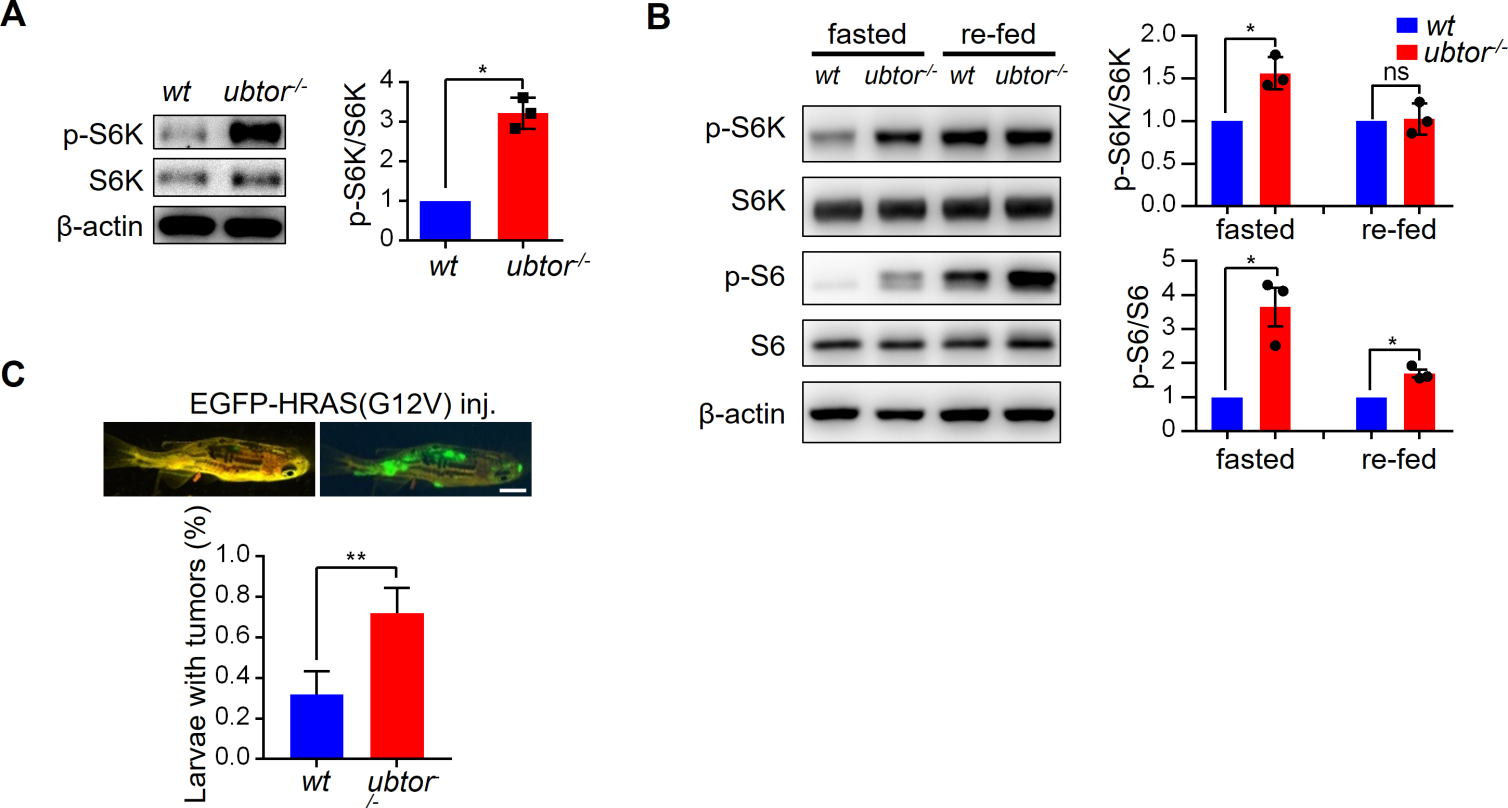
Disruption of *ubtor* gene increases mTOR signaling in zebrafish brain tissues. (**A**) Immunoblot analysis of p-S6K levels in wild type (*wt*) controls and *ubtor*^*-/-*^ mutants. The head tissues were isolated from 5 dpf zebrafish before introducing food to fish larvae. For quantitative analysis, the p-S6K/S6K values were normalized to that of *wt*. *t* = 9.840, *df* = 2, *P* = 0.0102. Representative results from three biological repeats. (**B**) Immunoblot analysis of mTOR signaling in *wt* controls and *ubtor*^*-/-*^ mutants under fasted and re-fed conditions. Zebrafish larvae of 13.5 dpf were starved for 36 hours, and then the larvae in the re-fed group were fed for 12 hours. Brain tissues were dissected out from the fasted and the re-fed larvae. For quantitative analysis, the p-S6K/S6K and p-S6/S6 values were normalized to that of *wt*. For p-S6K/S6K, *t* = 5.089, *df* = 2, *P* = 0.0385 under the fasted condition. For p-S6/S6, *t* = 4.637, *df* = 2, *P* = 0.0435 under the fasted condition, and *t* = 6.077, *df* = 2, *P* = 0.0260 under the re-fed condition. Representative results from three biological repeats. (**C**) Dominant-active HRAS(G12V) induced tumor formation in wild type controls (wt) and *ubtor* mutants. Three and four biological repeats for the *wt* and the mutant, respectively. *t* = 4.343, *df* = 5, *P* < 0.01. *n* = 59 and 37 for the *wt* and the mutant, respectively. *χ*_*1*_^*2*^ = 13.23, *P* < 0.001.

To determine if UBTOR’s regulation of mTOR signaling also occur in intact animals, we examined p-S6K levels in *ubtor* mutant and control larvae at 5 dpf, and found p-S6K was increased approximately 4-fold in the brains of *ubtor* mutant compared with the controls (Fig 7A). To further examine the effects of *ubtor* mutation on the mTOR signaling, zebrafish of 13.5 dpf were fasted for 12 hours to down-regulate, then refed with ample food for 12 hours to activate the mTOR activities. The brain tissues of fasted and fed zebrafish were analyzed for the levels of p-S6K and p-S6. Similar to results in rodents, food-deprivation reduced the levels of p-S6K and p-S6, and feeding restored those phosphorylation levels. The *ubtor* mutation caused significantly higher levels of p-S6K and p-S6 in the fasted animals, and significantly higher p-S6 levels in the refed animals (Fig 7B). Thus, *ubtor* mutation caused significant upregulation of the mTOR signaling in the intact animals.

HRAS(G12V), a dominant-active form of human oncogene *HRAS*, can promote tumor formation when overexpressed in zebrafish embryos [22]. Consistent with the in vitro effects of UBTOR knockdowns on cell growth and colony formation in the cultured cells, microinjection of the HRAS(G12V) construct into zebrafish *ubtor* mutant embryos increased neoplasia rate to over 70% compared with about 30% for the injection into the wild type controls (Fig 7C). In total, 19 out of the 59 wild type control zebrafish developed tumors, whilst 26 out of the 37 *ubtor* mutants had tumors (*χ*_*1*_^*2*^ = 13.23, *P* < 0.001).

**Fig 8:**
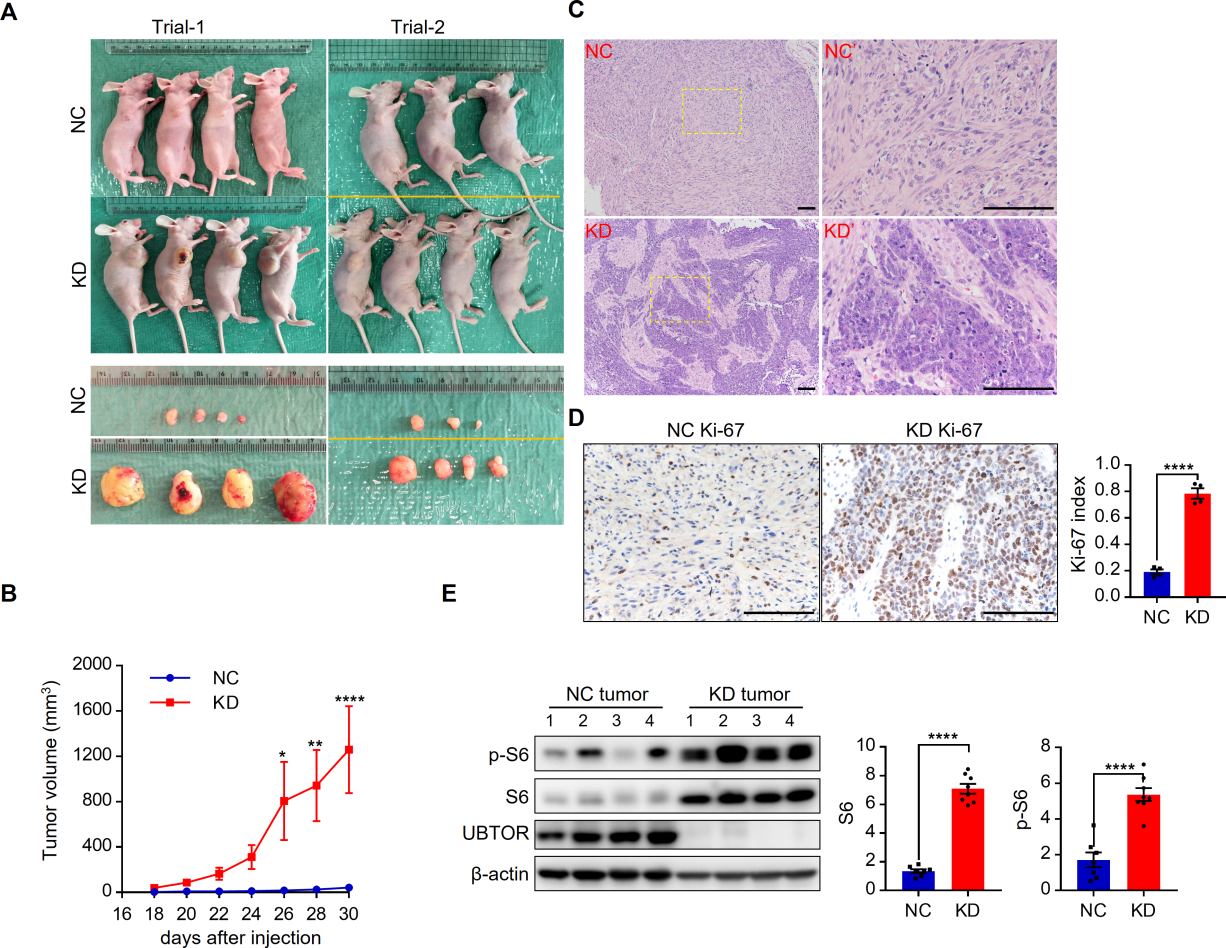
UBTOR depletion promotes xenograft tumor growth and mTOR signaling in nude mice. (**A**) Xenograft tumor growth in nude mice. A total of 1 x 10^6^ U87MG cells transfected with lentiviral shRNA (NC or UBTOR KD) were implanted into nude mice. At the endpoint, NC and KD tumors were removed and photographed. Results from two biological repeats (Trial-1 and Trial-2). (**B**) Quantitative analysis of the xenograft tumor volume growth. For the UBTOR-depletion KD factor, F _(1, 13)_ = 9.79, *P* = 0.008. Multiple comparison significance values are indicated on the graph. (**C**) Representative H&E staining images of the xenograft tumors. Right panels (NC’ and KD’) show enlarged views of the boxed area on the left panels. Scale bar, 100 μm. (**D**) Ki-67 staining of the xenograft tumors. *n* = 4 for the NC and the KD xenograft tumors, respectively. A minimum of 2000 nuclear profiles were counted per tumor lesion. *t* = 13.35, *df* = 6, *P* < 0.0001. Scale bar, 100 μm. (**E**) Immunoblot analysis of UBTOR, S6 and p-S6 expression levels in xenograft tumors. S6 and p-S6 levels were normalized to that of β-actin, and then to that of NC. For S6, *t* = 14.9, *df* = 13, *P* < 0.0001. For p-S6, *t* = 6.613, *df* = 13, *P* < 0.0001.

To further examine effects of UBTOR depletion on tumor growth in a mammalian model, we injected nude mice with U87MG cells. UBTOR-depleted U87MG cells formed significantly larger tumors than the control cells did in the nude mice (Fig 8A and 8B). Histopathology results showed UBTOR-depleted KD tumors were composed of pleomorphic cells featuring high cell density and high nuclear-cytoplasmic ratio (Fig 8C). The Ki-67 indexes were also significantly higher in the UBTOR-depleted KD tumors (Fig 8D). These features were consistent with high malignancy. Immunoblot results showed expression levels of S6 and p-S6 were elevated 4- and 3-fold in the UBTOR-depleted tumors (Fig 8E). Thus, UBTOR depletion promoted tumor growth and mTOR signaling in the xenograft mouse model.

## Discussion

In this study, UBTOR depletion promotes neurite and cellular growth, whilst UBTOR overexpression suppresses colony formation in cancer cell lines. Mechanistic studies in cultured cells revealed UBTOR stabilized DEPTOR, a component of the mTOR complexes, to inhibit the mTOR signaling. Investigations in the zebrafish model further showed disruption of *ubtor* gene upregulated the mTOR signaling and promoted HRAS(G12V) mediated tumor formation in intact animals. Similarly, UBTOR depletion promoted tumor growth and mTOR signaling in a xenograft mouse model. These findings demonstrate how Ubtor regulates cellular growth and neoplasia via mTOR signaling.

*Ubtor* is an unannotated gene and present in the vertebrate species only. It encodes a protein without any known functional domains despite of exhaustive sequence homology-based searches [23–25]. Ubtor protein is highly conserved in the vertebrates, with the shark protein shares 55% identities with the human protein. Searches of protein prediction servers [26–27] and protein-protein interaction databases [28] provided no clues into the function of Ubtor protein. No previous studies have indicated the function of the *Ubtor* gene or protein product, except that *UBTOR* gene was listed as one of the top down-regulated genes in adrenocortical adenoma [10], and a top-ranked mutated gene in pancreatic cancer [9, 11]. Expression analyses of human tumor samples also indicated *UBTOR* was markedly downregulated in adrenocortical cancer, pheochromocytoma and paraganglioma, and glioma. The present study highlights Ubtor’s functions in the cellular growth and the mTOR signaling. Abnormal activation of the mTOR signaling may provide explanation why *UBTOR* is downregulated or mutated in tumor tissues. Consistent with this view, our results show *UBTOR* knock-down promote growth in HEK293T and U87MG cells, and overexpression of *UBTOR* reduced colony formation in both the HEK293T cells and the carcinoma T24 cells. Enhancement of HRASG12V mediated tumor formation in the homozygous *ubtor* zebrafish mutant and larger xenograft tumor growth of UBOTR-depleted U87MG cells in the nude mice further support involvement of *Ubtor* in neoplasia. Interestingly, we show that NGF treatment in PC12 cells upregulate *Ubtor* expression. The induction of higher *Ubtor* expressions levels upon mTOR signaling activation may keep the mTOR signaling activity in check. Thus, *Ubtor* constitutes a novel negative feedback mechanism [29–31] to control mTOR signaling.

mTOR signaling activity is tightly regulated by various interacting proteins of the catalytic mTOR protein. DEPTOR is an integral component of both mTOC complexes and it functions an inhibitor of the mTOR signaling. Previous studies show that DEPTOR is ubiquitylated when mTOR signaling pathway is activated, which results in its degradation [6–8]. Our results show that UBTOR reduces ubiquitination of DEPTOR and stabilizes DEPTOR expression levels. Because the N terminal UBTOR^1-467^ binds to DEPTOR, a parsimonious model may indicate UBTOR is a deubiquitinating enzyme (DUB). Standard sequence-based homology searches fail to reveal an existence of DUB domain or any other functional domains in the UBTOR protein [23], neither do other specialized methods meant to detect remote homologies [24,25]. However, the primary sequences of DUB are diverse [32, 33]. Future studies will be required to determine if DUBTOR harbors deubiquitinating activity, or other mechanisms such as interference with the DEPTOR E3 ligase SCF [34] or trapping of mTOR complexes on the endoplasmic reticulum are involved.

In summary, our study provides insights into how *Ubtor* regulate cellular growth and mTOR signaling. Manipulations of Ubtor function may potentially be utilized to optimize mTOR signaling activities for treatments of cancers and other diseases.

## Materials and methods

### Ethics statement

The animal use protocols were reviewed and approved by the The Fudan University Shanghai Medical College Institutional Animal Care and Use Committee (110307–084, 130227–092, and 150119–088). All animals were handled in accordance with the NRC Guide for the Care and Use of Laboratory Animals and the Fudan University Regulations on Animal Experiments.

### Zebrafish husbandry

Zebrafish were maintained in a recirculating water system according to standard protocols [35]. Lines used in this study include AB, Tg(UAS:EGFP), Tg(*ubtor*:GAL4FF), and *ubtor* mutant. The wild type (*wt*) AB line was obtained from the University of Oregon Zebrafish Facility. The Tg(UAS:EGFP) line was a gift from K. Kawakami [36]. The Tg(*ubtor*:GAL4FF) line was obtained from an enhancer trap screen, and the *ubtor* mutant line was generated by targeted gene disruption, both using the AB line as the subject (see below).

### Enhancer-trap screen, transgenic mapping, gene names, and targeted gene disruption

The enhancer-trap vector used in this study contains minimal transposable elements of Tol2, a super core basal promoter, and coding sequence for a modified version of Gal4 transcriptional activator [37]. A mixture of the enhancer-trap vector and Tol2 transposase mRNA was microinjected into one-cell stage embryos. Transgenic founder fish were identified and selected based on EGFP expression patterns after crossing the injected fish with a Tg(UAS:EGFP) reporter line. Transgenic founder fish were outcrossed with the AB line for three generations before high-efficiency thermal asymmetric interlaced PCR [38, 39] was used to map the genomic insertion site of the enhancer trap vector. In the Tg(*ubtor*:GAL4FF) line, the enhancer-trap vector was mapped to the second intron of an uncharacterized gene on chromosome 7, which was subsequently named *ubtor* as results showed its protein product regulated protein ubiquitination and mTOR signaling. The mammalian Kiaa1024/Ubtor gene has two orthologs in the zebrafish genome. The trapped ortholog on chromosome 7 has a higher sequence identity to the mammalian sequences and was named *ubtora* (59% identity to the human protein sequence). The other ortholog on chromosome 18 was named *ubtorb* (26% identity to the human protein sequence). In situ hybridization results showed the *ubtorb* gene was transiently expressed in the Rohn-Beard cells between 24 and 72 hpf, and in a few cells in the trigeminal region after 72 hpf. Thus, it was unlikely to play significant roles in experiments in this study. To simplify the descriptions, the zebrafish *ubtora* gene was referred to as *ubtor* throughout this manuscript. To disrupt the *ubtor* gene in the zebrafish, three lesions were introduced into the zebrafish genome via the TALEN [40] and CRSPR/Cas9 mediated targeted gene modifications (see S1 Table for target site sequences and lesion information). This produced a 7-base deletion at position 22, a 10-base deletion at position 1319, and a 7-base insertion at position 1989 into the coding sequence of *ubtor*. These three frame-shift mutations introduced multiple stop codons to the Ubtor coding sequence. Thus, no functional protein product can be made in the homozygous mutant. This *ubtor* mutant line (*ubtor*^*d10*^) was outcrossed with the AB line for 5 generations and then used in this study.

### Constructs, antibodies, cell lines, primers, siRNAs, and shRNAs

The primer sequences, the target sequences and sources of siRNAs and Lentiviral shRNAs were listed in S1 Table. Plasmid constructs were generated via standard cloning methods. Construct descriptions, sources of antibodies and cell lines were listed in S2 Table.

### Operant conditioning

Operant conditioning was carried out using an apparatus built from a published design with modifications [41]. Briefly, four tanks of 13 cm x 5 cm x 5 cm (L x W x H) were placed on top of a LCD screen. Single zebrafish of 2 month old were placed into each tank with water 3 cm deep. During a 10 min adaptation period, a visual cue (blue checkerboard) was presented by the LCD screen on one side of each box for 10 sec and then on the other side for 10 sec. No electric shock was given during adaptation. During the conditioning period, the visual cue was presented on one side of each box. Free-moving zebrafish received a 0.2 sec 0.8V/cm electric shock whenever it entered the visual conditioned side of the tank and lingered for more than 0.5 sec. A 5 sec break period was provided after each shock, and the maximal number of shocks received by a fish within one minute period was limited to 3. The conditioning period lasted for 15 min, and two conditioning session were given, with the visual cue switching to the other side in the second session. Then the visual cue was removed for 30 min. For retrieval test, the CS visual cue was presented on one side of each box for 2 min. No electric shock was given during retrieval. During the entire test, a computer running MATLAB scripts took live images from an infra-red camera at 10 frames per second, tracked the fish movements, presented the visual cues, and delivered the electric shocks.

### C-start response

C-start response was carried out using an apparatus built from a published design with modifications [42]. Briefly, a 5 by 4 test grid was laser-cut onto a 70 mm x 50 mm x 2 mm (L x W x T) acrylic plate. A small vibrator rated at 12,000 rpm was fixed into the acrylic plate and driven by an Arduino micro-controller. The plate was placed on top of four steel balls and positioned by blocks on four corners. After each grid well was filled with single 5 dpf zebrafish, a 20–50 ms long current was sent to the vibrator to shake the plate and elicit C-starts. A high speed camera recorded the C-start response at 400–500 frames per second. Preliminary tests showed *ubtor* mutant fish exhibited reduced C-start after repetitive stimulations. Thus, ten repetitive stimuli were first given at 1 second intervals, then one minute later another stimulus was delivered and the C-start responses were recorded and analyzed.

### Primary hippocampal neuron culture

Primary hippocampal neurons were dissected from day 18 embryonic Sprague Dawley (SD) rats and cultured using a previously reported method [43]. Briefly, hippocampus was dissected out by fine forceps and digested in 0.05% trypsin-EDTA solution for 15 minutes in a 37 °C incubator. Digestion was stopped by DMEM/F12 medium with 10% FBS and neurons were collected by centrifuging at 1000 rpm for 8 minutes and re-suspended in DMEM/F12 medium with 10% FBS. Then neurons were plated on coverslips coated with poly-D-lysine at a density of 4 x 10^4^ cells/cm^2^. Twelve hours later, culture medium was replaced by neurobasal medium supplemented with 2% B27 (GIBCO, Life Technologies). siRNAs were transfected at the time of cell plating using Lipofectamine RNAiMAX (Thermos Scientific).

### Immunoblot analysis

Cells were lysed in 2 x SDS sample buffer (100 mM Tris-HCl pH 6.8, 4% SDS, 0.02% bromophenol blue, 20% glycerol and 200 mM DTT) then boiled at 95°C for 10 min. Proteins in cell lysates were resolved by 6%-20% SDS-PAGE, and tank-transferred onto nitrocellulose membranes. Membranes were probed with the primary antibodies overnight and detected with HRP conjugated secondary antibodies (see S2 Table). Immuno-signals were developed by enhanced chemiluminescence, recorded by a FluorChem E system (ProteinSimple), and analyzed by ImageJ.

### Immunoprecipitation and pulldown assays

For immunoprecipitation, cell lysates were incubated with anti-FLAG-M2 beads (Sigma-Aldrich) or anti-HA-beads (Sigma-Aldrich) at 4°C for 4 hours, and then the beads were washed with lysis buffer for 3 times. Finally, the beads were mixed with 2 x SDS sample buffer (100 mM Tris-HCl, pH 6.8, 4% SDS, 0.02% bromophenol blue 20% glycerol and 200 mM DTT) and boiled at 95°C for 10 minutes. For pull down assays, cell lysates and GST-tagged proteins purified from bacteria were mixed with glutathione Sepharose beads (GE Life Sciences) at 4°C for 4 hours. Beads were washed 3 times with lysis buffer and boiled with 2 x SDS sample buffer at 95°C for 10 minutes.

### GST and His tagged protein purification

Bacteria BL21(DE3) expressing GST-tagged or His-tagged proteins were incubated with bacterium lysis buffer (20 mM Tris-Cl, pH 8.0, 500 mM NaCl, 1 mM DTT, 5% glycerol, 1% TritonX-100, protease inhibitor cocktail, 300 ug/ml lysozyme) at 4°C for 1 hour then disrupted with sonication. The lysates were cleared by centrifugation and then incubated with glutathione Sepharose or Ni-NTA Agarose beads at 4°C for 1 hour. For GST-tagged proteins, the beads were washed three times with the lysis buffer and the GST-tagged proteins were eluted with elution buffer (50 mM Tris-Cl, pH 8, 150 mM NaCl, 1 mM DTT, 5% glycerol, 1% TritonX-100) containing 10 mM glutathione. For His-tagged protein, the beads were washed three times with the lysis buffer containing 20 mM imidazole and the His-tagged proteins were eluted with elution buffer containing 500 mM imidazole.

### Cell proliferation assay

Cells were plated at 1500 cells/well in 96-well plate. The substrate WST-8 from the CCK-8 kit (Dojindo) was added from 6 hours to 4 days post plating and incubated for 4 hours, followed by absorbance measurement at 450 nm.

### Colony formation assay

Cells were plated at 2 x 10^4^ (HEK293T) or 5 x 10^4^ (T24) cells/dish in a 6 cm diameter petri-dish and then transfected with plasmid constructs as indicated. Puromycin was added to the culture medium at 1 μg/ml and then the cells were fixed and stained with 0.01% crystal violet after two weeks.

### HRAS(G12V) induced tumor formation

A Tol2-pCMV-GFP-HRAS(G12V) plasmid mixed with Tol2 transposase mRNA was injected to zebrafish embryos at one cell stage. Fish of one month old were examined for neoplasia by fluorescence microscopy and paraffin section of the tumors.

### Xenograft in nude mice

To generate xenograft tumors, a total of 1x10^6^ U87MG cells transfected with lentivirus negative control (NC) or UBTOR Lenti-1 shRNAs were suspended in 100 μl of MEM medium without serum and implanted subcutaneously into the flanks of 7-week-old female BALB/c nude mice. The tumor volume was measured every other days by a digital caliper. At the end point, portions of the xenograft tumors were fixed with 4% paraformaldehyde, paraffin embedded, sectioned, and subjected to hematoxylin/eosin and Ki-67 staining. The remaining tumor tissues were processed for protein extraction and subsequent immunoblot analyses.

### Immunofluorescence analysis

Cells were briefly washed with PBS and fixed with 4% paraformaldehyde for 10 minutes at room temperature. Fixed cells were washed with PBS and then post-fixed with methanol at -20°C for 15 minutes. After permeabilization with 0.5% TritonX-100 in PBS for 7 min, cells were blocked with blocking solution (PBS containing 2% sheep serum, 2% goat serum 0.2% BSA and 0.1% Tween-20) and incubated overnight at 4°C with primary antibodies diluted in blocking solution. Cells were then washed with PBS containing 0.1% Tween-20 and incubated with secondary antibodies (see S1 Table) for 2 hours at room temperature. Pearson’s correlation coefficients were measured using ImageJ package Fiji.

### Statistical analysis

Data are expressed as mean ± sem. Significance values are denoted as: *: *P* < 0.05, **: *P* < 0.01, ***: *P* < 0.001, and ****: *P* < 0.0001. Sample sizes for each figure are given in the figure legends. All quantified data are representative of at least three biological repeats. Significance of differences was assessed by two-tailed Student’s *t-* test, one sample *t*-test, or two-way ANOVA analysis when appropriate.

## Supporting information

S1 Fig *ubtor/kiaa1024* gene mapping and expression. (**A**) Transgenic expression of EGFP in a 5 dpf larva from the cross between the enhancer trap line Tg(*ubtor*:GAL4FF) and the reporter line Tg(UAS:EGFP). Larva was embedded in agarose and imaged with a confocal microscope. Five image fields were stitched together to show the full length of the animal. (**B**) Genomic sequences at the insertion site in the Tg(*ubtor*:GAL4FF) line. The Tol2-GAL4FF transgene is inserted at the second intron in the *ubtor* gene. (**C**) Phylogeny tree of *Ubtor* genes in various vertebrate species. No *Ubtor* homologs were found outside the vertebrates. (**D**) EGFP expression in the brain region in the *ubtor* enhancer trap line. Ha: habenula. Dorsal View. (**E-F**) In situ hybridization analysis of endogenous *ubtor* gene expressions at 3 dpf and 5 dpf. Dorsal View. Scale bars, 200 μm.
(TIF)

S2 Fig Effects of Ubtor depletion in cultured cells. (**A**) Neurite outgrowth in primary culture of rat hippocampal neurons at 56 HIV. Dissociated hippocampal neurons were transfected with either negative control siRNA (NC) or *Ubtor* siRNA (Ubtor siKD) and then cultured in vitro for 56 hrs. Neurites were stained by the acetylated tubulin antibody. Transfected cells were indicated by the Cy3 fluorescence signals from the Cy3-labeled siRNAs. Scale bar, 50 μm. Quantitative analysis of neurite outgrowth at 56 HIV is shown on the right. Neurite lengths were measured from 10 images for the NC, and 10 images for the Ubtor siKD groups, taken from 3 independent experiments. n = 204 for NC, and n = 220 for Ubtor siKD groups. *t* = 8.837, *df* = 422, *P* < 0.0001. (**B**) NGF-induced neurite outgrowths in the PC12 cells transfected with either negative control siRNA (NC) or *Ubtor* siRNA (Ubtor siKD). Transfected cells were serum-starved overnight and treated with 50 ng/ml of NGF for 0 and 48 hours. Scale bar, 20 μm. Neurite outgrowth rates were calculated from 6 images for the NC, and 5 images for the Ubtor siKD groups, taken from 3 independent experiments. *t* = 5.927, *df* = 9, *P* < 0.001. Neurite lengths of differentiated cells were measured in these images. *n* = 224 and 288 for the NC and the Ubtor siKD group, respectively. *t* = 15.72, *df* = 510, *P* < 0.0001. (**C**) Cy3-siRNA transfected cells. The fluorescence signals from Cy3- siRNA indicate essentially all cells were transfected. (**D**) qRT-PCR analysis of Ubtor expression levels in the original PC12 cells and the ld-PC12 cells. Expression levels relative to GAPDH levels are normalized to the original PC12 group. Three biological repeats. *t* = 29.16, *df* = 4, *P* < 0.0001.
(TIF)

S3 Fig Expression analyses of *UBTOR* levels in human tumor tissues. (**A**) *UBTOR* expression levels were significantly down-regulated in adrenocortical cancer samples. Graph was generated by the Xena Browser, comparing the TCGA Adrenocotrical Cancer samples with the GTEX Adrenal Gland samples. (**B**) *UBTOR* expression levels were decreased in pheochromocytoma and paraganglioma (PCPG), and glioma (GBM and GBMLGG) cancer samples. Graph was generated by the FireBrowse Server using the TCGA tumor and control samples.
(TIF)

S4 Fig Immunoblot analysis of signaling pathways in the PC12 and HEK293T cells. (**A**) Immunoblot analysis of mTOR signalling pathway in the PC12 cells transfected with either negative control siRNA (NC) or *Ubtor* siRNA (Ubtor siKD). Transfected cells were serum starved overnight and treated with 50 ng/ml of NGF for 0 to 24 hours. In addition, cells were treated with 100 nM of rapamycin (rapa) or vehicle (DMSO) for 30 min after 24 hours of NGF treatment. GAPDH was used as a loading control. Quantitative analysis of p-S6 levels is shown on the right. Four biological repeats. Statistics significance values are indicated on the graph. (**B**) Immunoblot analysis of p-ERK1/2 levels in the PC12 cells. Transfected cells were treated as in **A**. Representative results from 3 biological repeats. Quantitative analysis of the immunoblots is shown below. (**C**) Immunoblot analysis of p-ERK1/2 levels in HEK293T cells. Transfected cells were serum starved overnight and then treated with 20% FBS for indicated time. Representative results from 3 biological repeats. Quantitative analysis of the immunoblots is shown below.
(TIF)

S5 Fig Orientation of UBTOR on the cellular membrane. Schematic cartoon on top shows the predicated transmembrane domain (in red) located at the carboxyl terminus of UBTOR. Live HEK293T cells expressing UBTOR tagged with EGFP at the carboxyl end (UBTOR^EGFP^) or the amino terminal (^EGFP^UBTOR) were reacted in suspension with anti-GFP antibody, and then washed with PBS, fixed, and stained with secondary antibody (in red). Scale bar, 10 μm.
(TIF)

S6 Fig Validation of the mTOR antibody. (**A**) Immunofluorescence signal was reduced by siRNA mediated knock-down of mTOR protein. HeLa cells were transfected with either Cy3 dye labeled negative control siRNA (NC) or *mTOR* siRNA (mTOR siKD) and then stained with the antibody against mTOR. Quantification result is shown on the right. *t* = 16.86, *df* = 337, *P* < 0.0001. (**B**) Immunoblot analysis of the specificity of the mTOR antibody. HeLa cells were transfected with either negative control siRNA (NC) or *mTOR* siRNA (mTOR siKD) and then immunoblotted with the mTOR antibody. Quantification result is shown on the right. *t* = 18.85, *df* = 2, *P* < 0.01.
(TIF)

S7 Fig Effects of *ubtor* gene disruption in the zebrafish. (**A**) *ubtor* gene disruption enhances freezing in operant conditioning tests. Data from three biological repeats. *n* = 14 and 12 for the wild type (*wt*) controls and the *ubtor*^*-/-*^ mutants, respectively. For the genotype factor, F_(1, 24)_ = 15.62, *P* < 0.001. Multiple comparison significance values are indicated on the graph. See Methods for test procedure. (**B**) *ubtor* gene disruption decreases vibration induced C-start responses. Data from three biological repeats. *n* = 144 and 132 for the *wt* controls and the *ubtor*^*-/-*^ mutants, respectively. *χ*_*1*_^*2*^ = 10.9, *P* < 0.001. See Methods for test procedure.
(TIF)

S1 Table Primer sequences, gene targeting sequences and sources of siRNAs and Lentiviral shRNAs. (XLSX)

S2 Table Construct descriptions, sources of antibodies and cell lines. (XLSX)
