# Supplementary figures and images for: UBTOR/KIAA1024 regulates neurite outgrowth and neoplasia through mTOR signaling
Source: PLoS Genet. 2018 Aug 6;14(8):e1007583. doi: 10.1371/journal.pgen.1007583 (PMC6095612; doi:10.1371/journal.pgen.1007583)

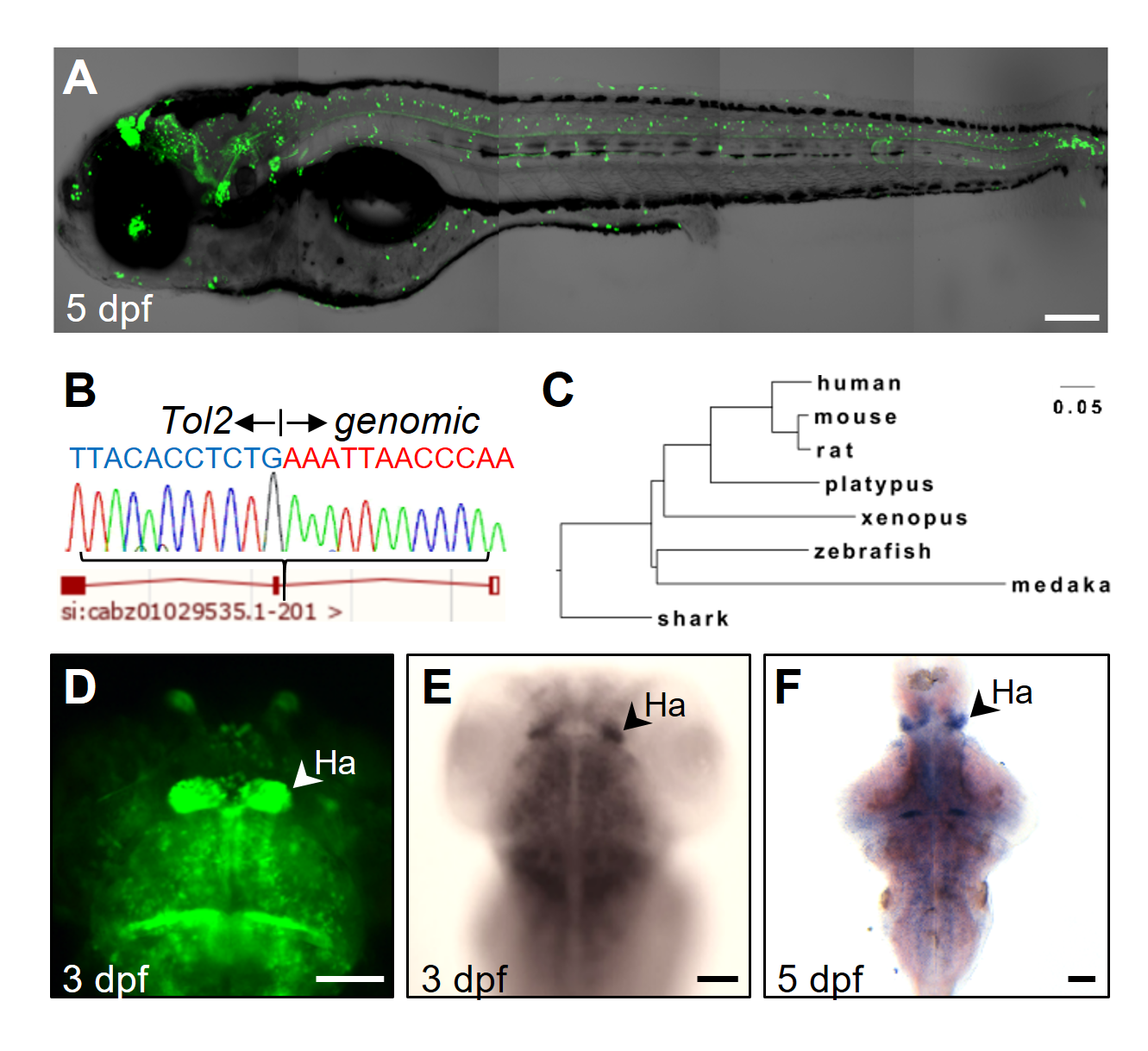

Supplement: S1 Fig — (A) Transgenic expression of EGFP in a 5 dpf larva from the cross between the enhancer trap line Tg(ubtor:GAL4FF) and the reporter line Tg(UAS:EGFP). Larva was embedded in agarose and imaged with a confocal microscope. Five image fields were stitched together to show the full length of the animal. (B) Genomic sequences at the insertion site in the Tg(ubtor:GAL4FF) line. The Tol2-GAL4FF transgene is inserted at the second intron in the ubtor gene. (C) Phylogeny tree of Ubtor genes in various vertebrate species. No Ubtor homologs were found outside the vertebrates. (D) EGFP expression in the brain region in the ubtor enhancer trap line. Ha: habenula. Dorsal View. (E-F) In situ hybridization analysis of endogenous ubtor gene expressions at 3 dpf and 5 dpf. Dorsal View. Scale bars, 200 μm. (TIF) [file pgen.1007583.s001.TIF]

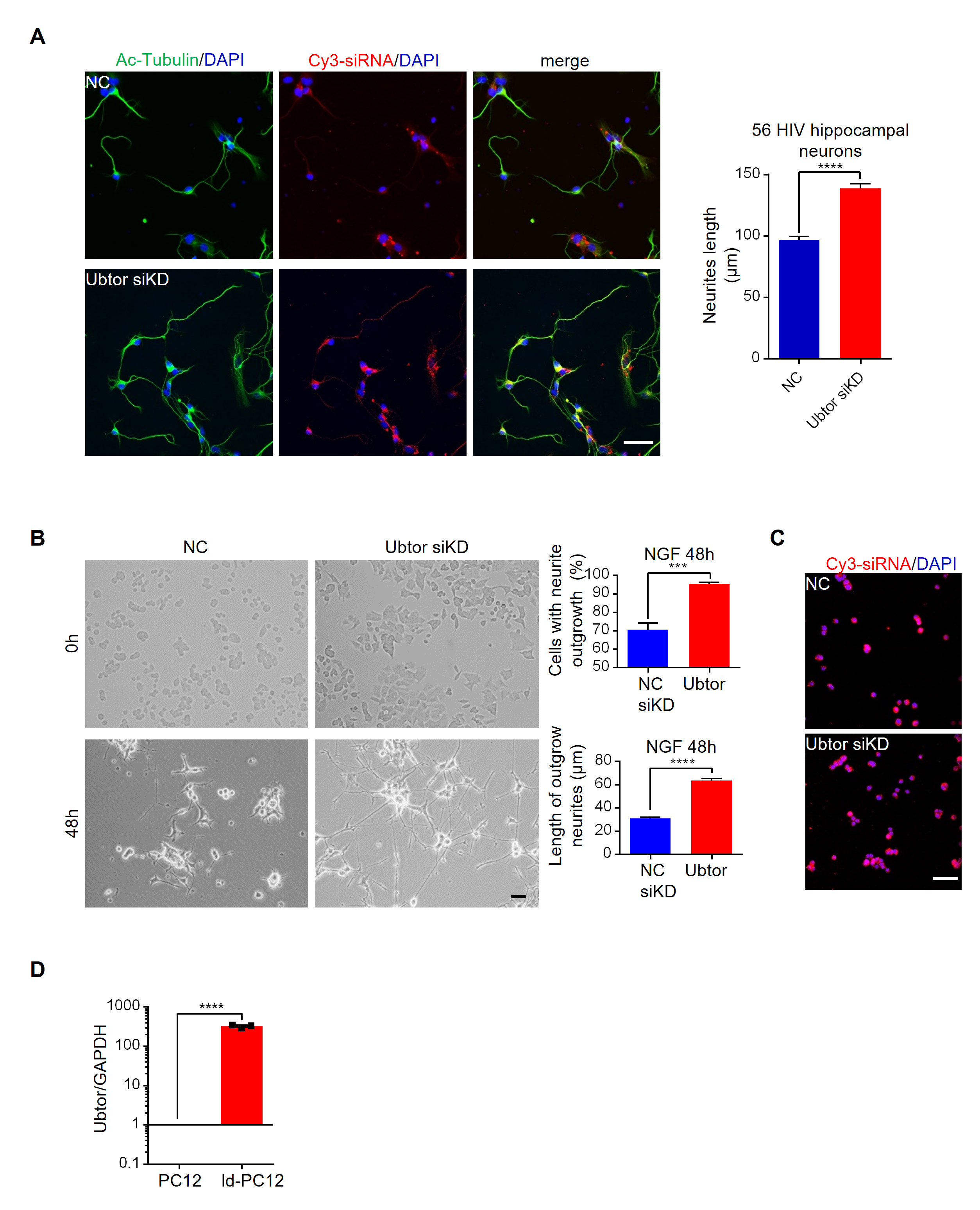

Supplement: S2 Fig — (A) Neurite outgrowth in primary culture of rat hippocampal neurons at 56 HIV. Dissociated hippocampal neurons were transfected with either negative control siRNA (NC) or Ubtor siRNA (Ubtor siKD) and then cultured in vitro for 56 hrs. Neurites were stained by the acetylated tubulin antibody. Transfected cells were indicated by the Cy3 fluorescence signals from the Cy3-labeled siRNAs. Scale bar, 50 μm. Quantitative analysis of neurite outgrowth at 56 HIV is shown on the right. Neurite lengths were measured from 10 images for the NC, and 10 images for the Ubtor siKD groups, taken from 3 independent experiments. n = 204 for NC, and n = 220 for Ubtor siKD groups. t = 8.837, df = 422, P < 0.0001. (B) NGF-induced neurite outgrowths in the PC12 cells transfected with either negative control siRNA (NC) or Ubtor siRNA (Ubtor siKD). Transfected cells were serum-starved overnight and treated with 50 ng/ml of NGF for 0 and 48 hours. Scale bar, 20 μm. Neurite outgrowth rates were calculated from 6 images for the NC, and 5 images for the Ubtor siKD groups, taken from 3 independent experiments. t = 5.927, df = 9, P < 0.001. Neurite lengths of differentiated cells were measured in these images. n = 224 and 288 for the NC and the Ubtor siKD group, respectively. t = 15.72, df = 510, P < 0.0001. (C) Cy3-siRNA transfected cells. The fluorescence signals from Cy3- siRNA indicate essentially all cells were transfected. (D) qRT-PCR analysis of Ubtor expression levels in the original PC12 cells and the ld-PC12 cells. Expression levels relative to GAPDH levels are normalized to the original PC12 group. Three biological repeats. t = 29.16, df = 4, P < 0.0001. (TIF) [file pgen.1007583.s002.TIF]

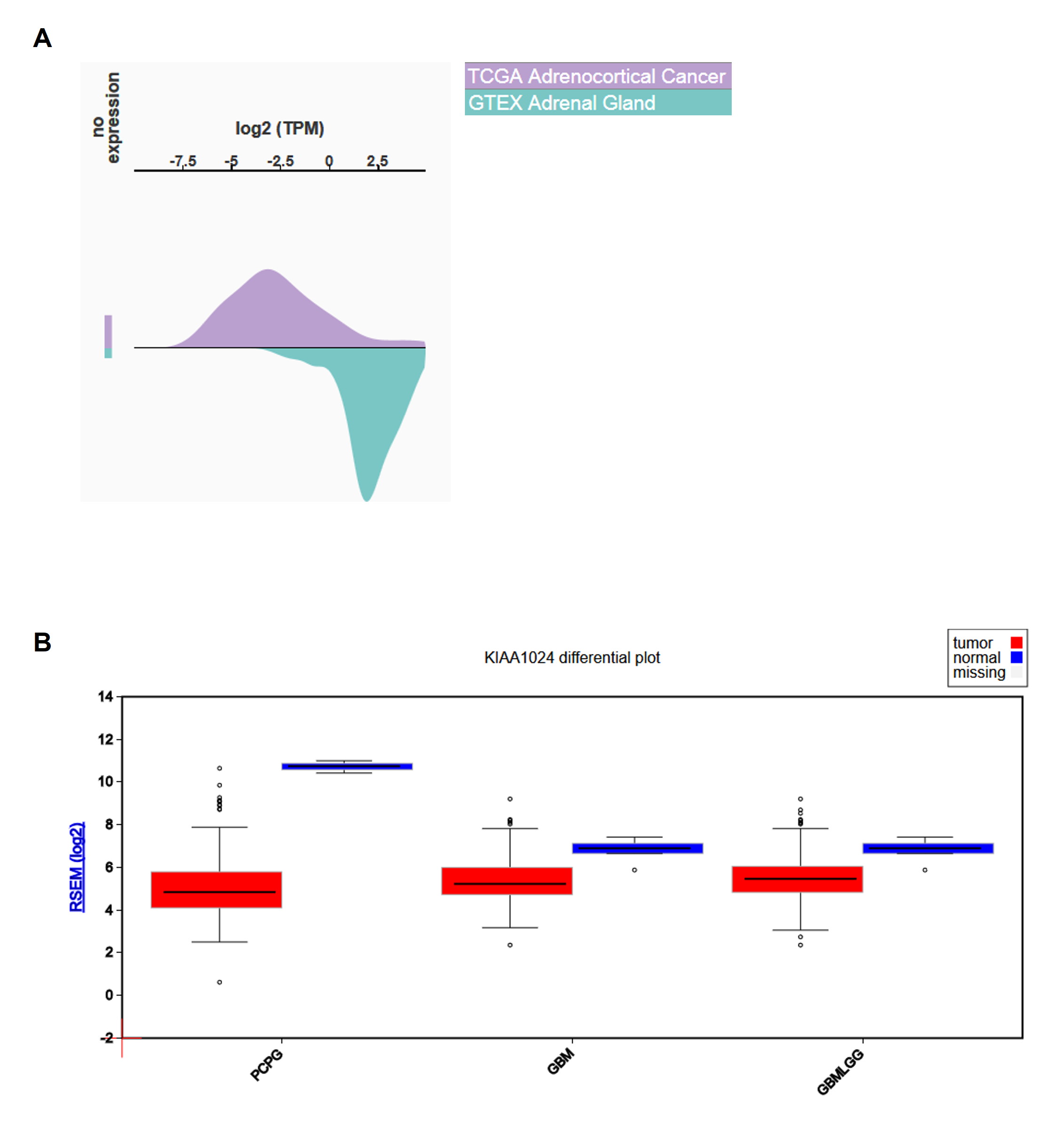

Supplement: S3 Fig — (A) UBTOR expression levels were significantly down-regulated in adrenocortical cancer samples. Graph was generated by the Xena Browser, comparing the TCGA Adrenocotrical Cancer samples with the GTEX Adrenal Gland samples. (B) UBTOR expression levels were decreased in pheochromocytoma and paraganglioma (PCPG), and glioma (GBM and GBMLGG) cancer samples. Graph was generated by the FireBrowse Server using the TCGA tumor and control samples. (TIF) [file pgen.1007583.s003.TIF]

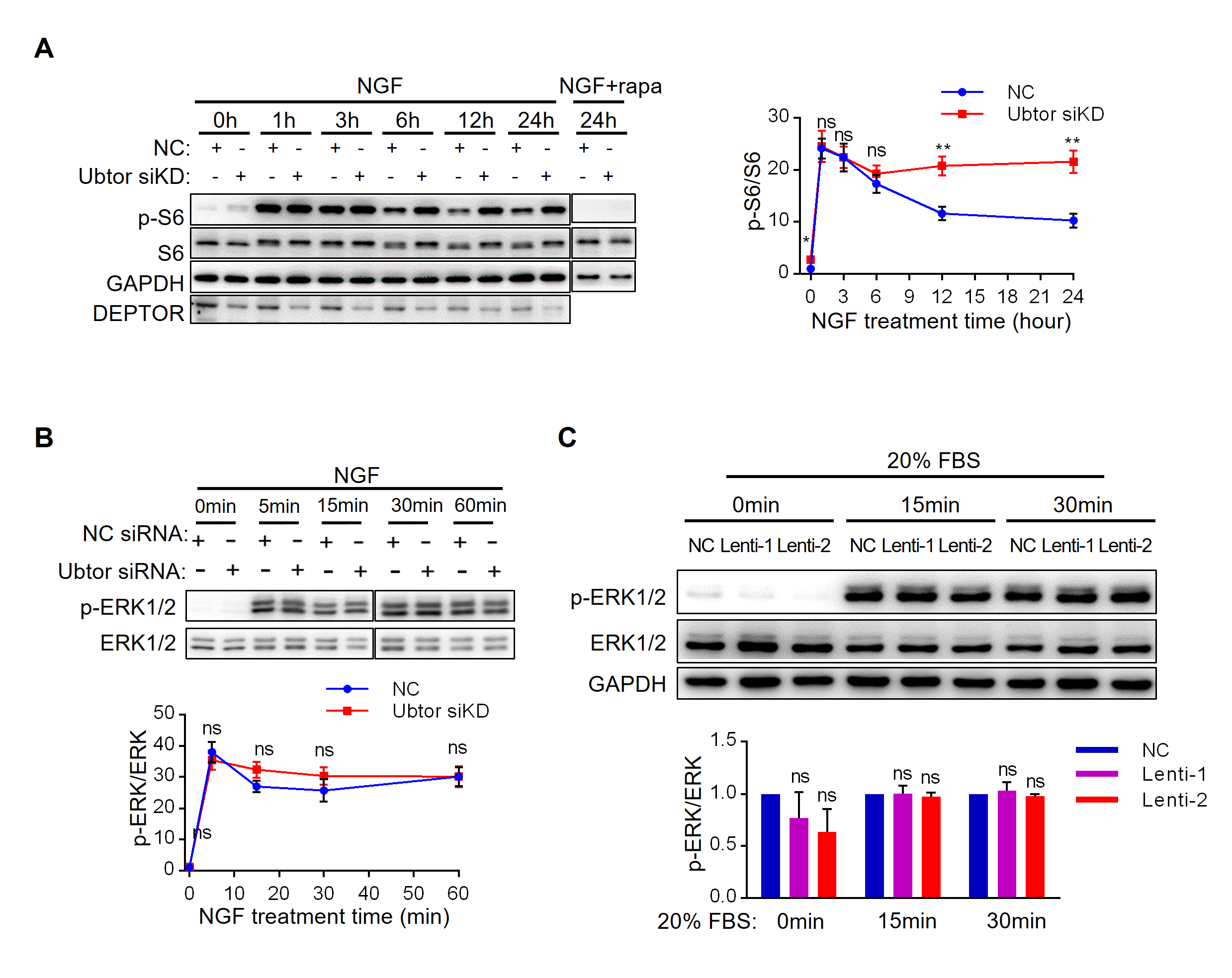

Supplement: S4 Fig — (A) Immunoblot analysis of mTOR signalling pathway in the PC12 cells transfected with either negative control siRNA (NC) or Ubtor siRNA (Ubtor siKD). Transfected cells were serum starved overnight and treated with 50 ng/ml of NGF for 0 to 24 hours. In addition, cells were treated with 100 nM of rapamycin (rapa) or vehicle (DMSO) for 30 min after 24 hours of NGF treatment. GAPDH was used as a loading control. Quantitative analysis of p-S6 levels is shown on the right. Four biological repeats. Statistics significance values are indicated on the graph. (B) Immunoblot analysis of p-ERK1/2 levels in the PC12 cells. Transfected cells were treated as in A. Representative results from 3 biological repeats. Quantitative analysis of the immunoblots is shown below. (C) Immunoblot analysis of p-ERK1/2 levels in HEK293T cells. Transfected cells were serum starved overnight and then treated with 20% FBS for indicated time. Representative results from 3 biological repeats. Quantitative analysis of the immunoblots is shown below. (TIF) [file pgen.1007583.s004.TIF]

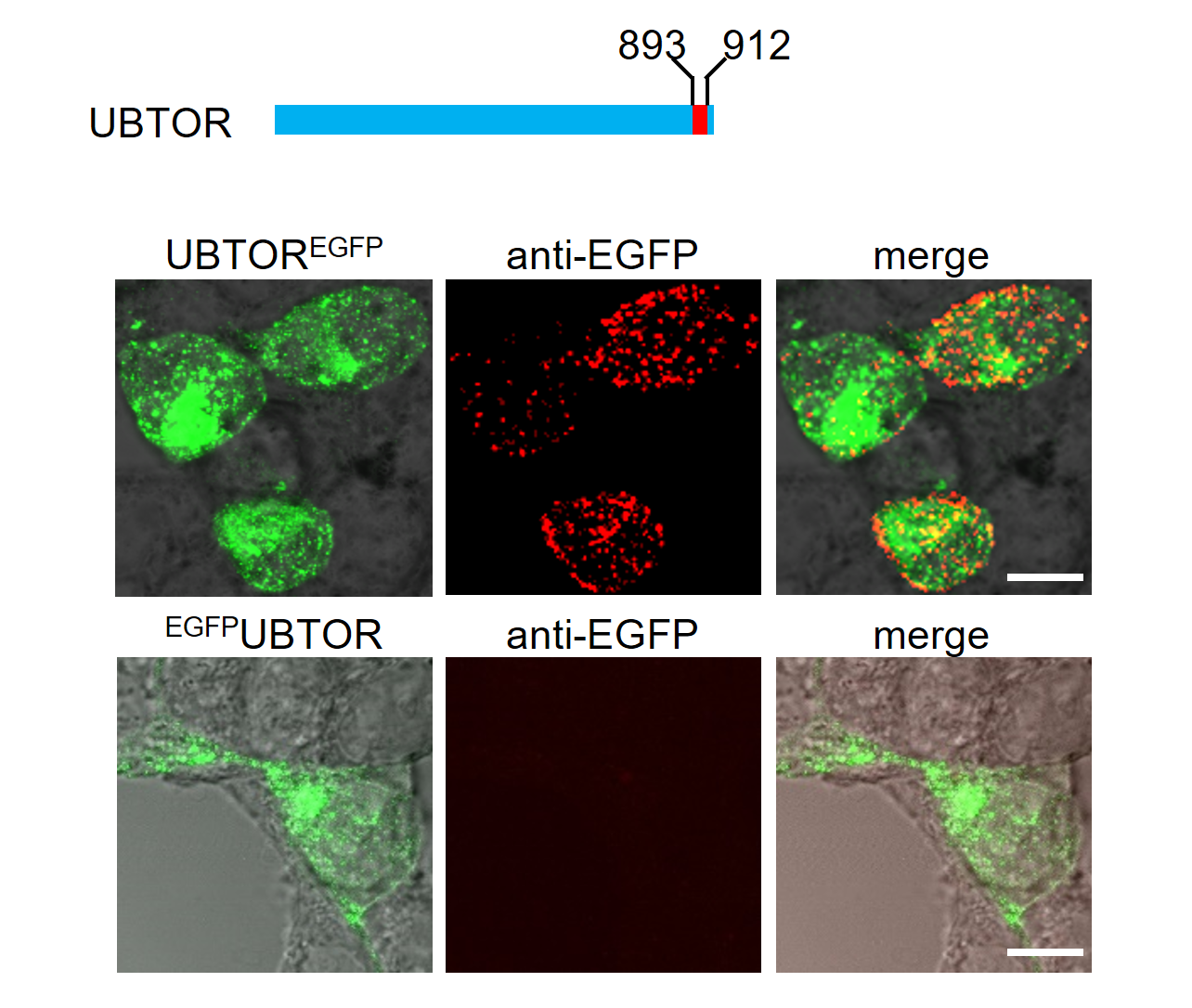

Supplement: S5 Fig — Schematic cartoon on top shows the predicated transmembrane domain (in red) located at the carboxyl terminus of UBTOR. Live HEK293T cells expressing UBTOR tagged with EGFP at the carboxyl end (UBTOREGFP) or the amino terminal (EGFPUBTOR) were reacted in suspension with anti-GFP antibody, and then washed with PBS, fixed, and stained with secondary antibody (in red). Scale bar, 10 μm. (TIF) [file pgen.1007583.s005.TIF]

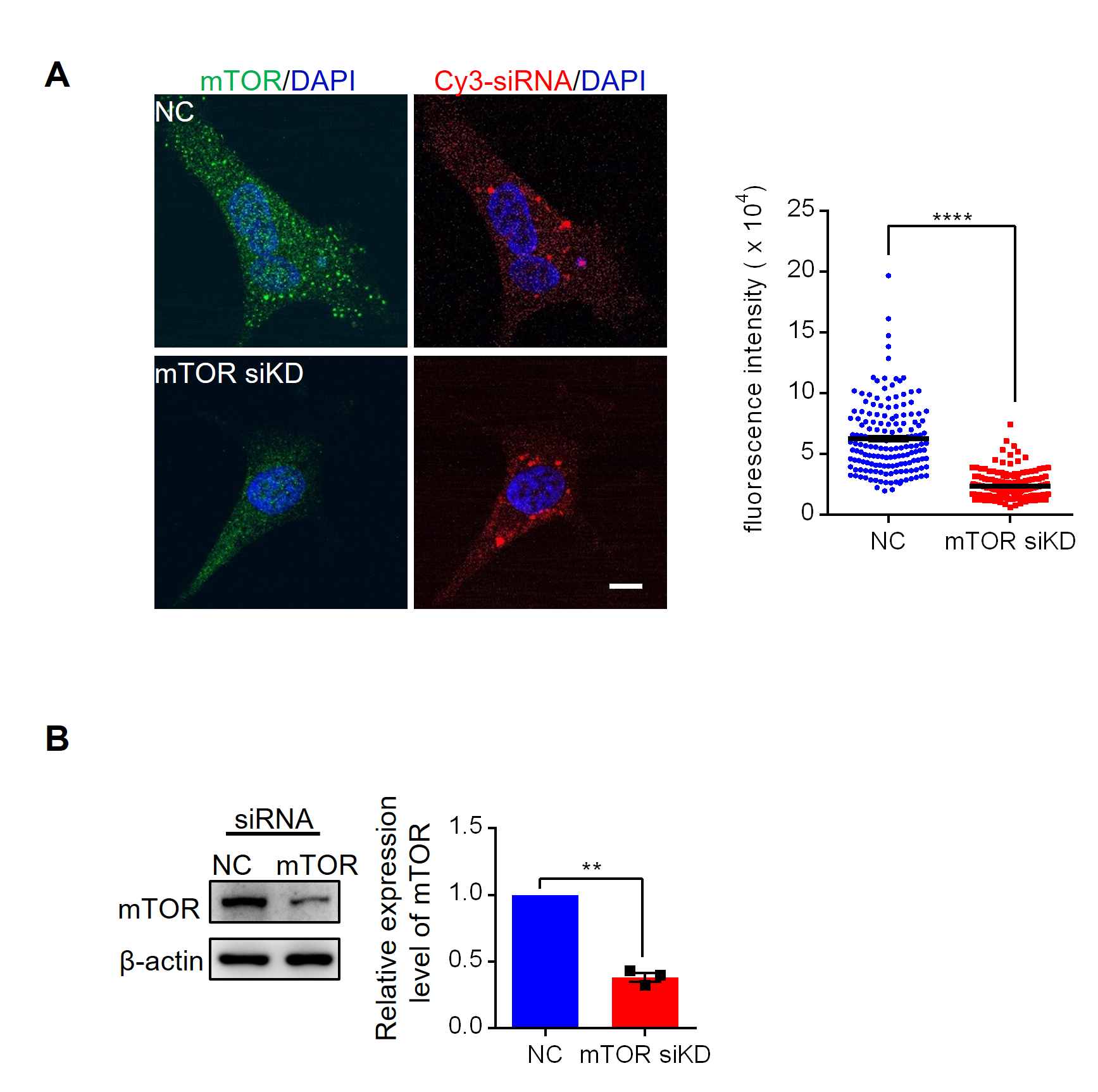

Supplement: S6 Fig — (A) Immunofluorescence signal was reduced by siRNA mediated knock-down of mTOR protein. HeLa cells were transfected with either Cy3 dye labeled negative control siRNA (NC) or mTOR siRNA (mTOR siKD) and then stained with the antibody against mTOR. Quantification result is shown on the right. t = 16.86, df = 337, P < 0.0001. (B) Immunoblot analysis of the specificity of the mTOR antibody. HeLa cells were transfected with either negative control siRNA (NC) or mTOR siRNA (mTOR siKD) and then immunoblotted with the mTOR antibody. Quantification result is shown on the right. t = 18.85, df = 2, P < 0.01. (TIF) [file pgen.1007583.s006.TIF]

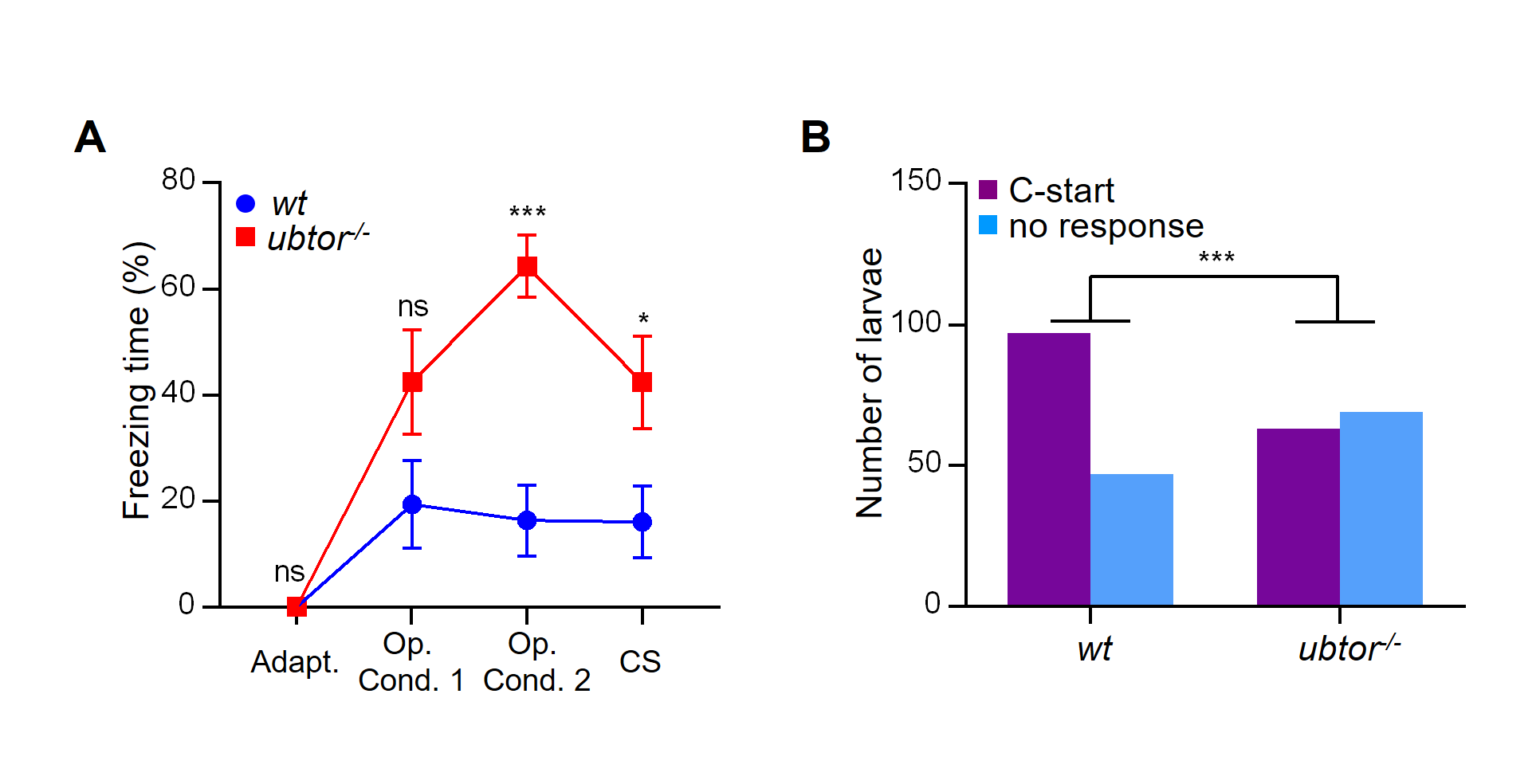

Supplement: S7 Fig — (A) ubtor gene disruption enhances freezing in operant conditioning tests. Data from three biological repeats. n = 14 and 12 for the wild type (wt) controls and the ubtor-/- mutants, respectively. For the genotype factor, F(1, 24) = 15.62, P < 0.001. Multiple comparison significance values are indicated on the graph. See Methods for test procedure. (B) ubtor gene disruption decreases vibration induced C-start responses. Data from three biological repeats. n = 144 and 132 for the wt controls and the ubtor-/- mutants, respectively. χ12 = 10.9, P < 0.001. See Methods for test procedure. (TIF) [file pgen.1007583.s007.TIF]
